# Provitamin A biofortification of cassava enhances shelf life but reduces dry matter content of storage roots due to altered carbon partitioning into starch

**DOI:** 10.1111/pbi.12862

**Published:** 2017-12-27

**Authors:** Getu Beyene, Felix R. Solomon, Raj D. Chauhan, Eliana Gaitán‐Solis, Narayanan Narayanan, Jackson Gehan, Dimuth Siritunga, Robyn L. Stevens, John Jifon, Joyce Van Eck, Edward Linsler, Malia Gehan, Muhammad Ilyas, Martin Fregene, Richard T. Sayre, Paul Anderson, Nigel J. Taylor, Edgar B. Cahoon

**Affiliations:** ^1^ Donald Danforth Plant Science Center St. Louis MO USA; ^2^ Center for Plant Science Innovation Department of Biochemistry E318 Beadle Center University of Nebraska‐Lincoln Lincoln NE USA; ^3^ Department of Biology University of Puerto Rico Mayaguez Puerto Rico; ^4^ Department of Horticultural Sciences Texas A&M AgriLife Research and Extension Center Weslaco TX USA; ^5^ Boyce Thompson Institute Ithaca NY USA; ^6^ New Mexico Consortium Los Alamos National Laboratory Los Alamos NM USA; ^7^ Present address: University of Arizona Tucson AZ USA; ^8^ Present address: African Development Bank Abidjan 01 Côte d'Ivoire

**Keywords:** cassava, dry matter, fatty acid, starch, β‐carotene, provitamin A

## Abstract

Storage roots of cassava (*Manihot esculenta* Crantz), a major subsistence crop of sub‐Saharan Africa, are calorie rich but deficient in essential micronutrients, including provitamin A β‐carotene. In this study, β‐carotene concentrations in cassava storage roots were enhanced by co‐expression of transgenes for deoxy‐d‐xylulose‐5‐phosphate synthase (*DXS*) and bacterial phytoene synthase (*crtB*), mediated by the patatin‐type 1 promoter. Storage roots harvested from field‐grown plants accumulated carotenoids to ≤50 μg/g DW, 15‐ to 20‐fold increases relative to roots from nontransgenic plants. Approximately 85%–90% of these carotenoids accumulated as all‐*trans*‐β‐carotene, the most nutritionally efficacious carotenoid. β‐Carotene‐accumulating storage roots displayed delayed onset of postharvest physiological deterioration, a major constraint limiting utilization of cassava products. Large metabolite changes were detected in β‐carotene‐enhanced storage roots. Most significantly, an inverse correlation was observed between β‐carotene and dry matter content, with reductions of 50%–60% of dry matter content in the highest carotenoid‐accumulating storage roots of different cultivars. Further analysis confirmed a concomitant reduction in starch content and increased levels of total fatty acids, triacylglycerols, soluble sugars and abscisic acid. Potato engineered to co‐express *DXS* and *crtB* displayed a similar correlation between β‐carotene accumulation, reduced dry matter and starch content and elevated oil and soluble sugars in tubers. Transcriptome analyses revealed a reduced expression of genes involved in starch biosynthesis including ADP‐glucose pyrophosphorylase genes in transgenic, carotene‐accumulating cassava roots relative to nontransgenic roots. These findings highlight unintended metabolic consequences of provitamin A biofortification of starch‐rich organs and point to strategies for redirecting metabolic flux to restore starch production.

## Introduction

Cassava (*Manihot esculenta* Crantz) is a starchy root crop widely grown in Southeast Asia, Latin America, the Caribbean and sub‐Saharan Africa (SSA) for human and livestock consumption, and as a feedstock for biofuels and other bio‐based materials (Howeler *et al*., 2013). Cassava ranks first among crops in volume of production (146.8 million tonnes) in Africa accounting for over 50% of the world total production in 2014 (FAOSTAT, 2017). In SSA, production is dominated by small‐scale subsistence farmers in low‐input cropping systems. Cassava is well adapted to subsistence farming systems because of its ease of propagation from stem cuttings and its ability to thrive in low‐input, stress‐prone environments (El‐Sharkawy, 2004; Howeler *et al*., 2013). Cassava storage roots are an excellent source of dietary calories due to the high content of starch (Montagnac *et al*., 2009; Rickard *et al*., 1991). Despite these desirable qualities, cassava production in SSA is limited by its susceptibility to disease (Bull *et al*., 2011; Reynolds *et al*., 2015) and to rapid postharvest physiological deterioration (PPD) (Naziri *et al*., 2014; Wenham, 1995). The latter restricts the crop as source of income for subsistence farmers due to difficulties in transporting rural‐grown cassava to urban markets and factory locations. Although cassava storage roots are good sources of calories, they have low concentrations of many essential nutrients including iron, zinc, protein and provitamin A (Sayre *et al*., 2011). Heavy reliance on high‐calorie, low‐nutrient foods such as cassava is a contributing factor to widespread mineral and vitamin deficiencies in SSA, especially in children. Strategies for correcting mineral and vitamin deficiencies using supplements have had little success in part because they are short‐lived. Biofortification of staple crops such as cassava has considerable potential for long‐term impacts in correcting deficiencies of critically essential vitamins and minerals.

Enhancement of provitamin A carotenoids is an important nutritional target for the improvement of crops such as cassava, rice, banana and other staple food crops in developing countries (Bouis and Saltzman, 2017; Mayer *et al*., 2008; Sayre *et al*., 2011). Vitamin A deficiency (VAD) is widespread in low‐income countries in the tropics (WHO, 2009) and is particularly prevalent in SSA. In Nigeria, the world's largest producer of cassava, 83% of the children aged 2–5 years were found to exhibit VAD (Maziya‐Dixon *et al*., 2004). Among the outcomes of chronic VAD are blindness, which affects 250 000–500 000 children annually, and susceptibility to infections (WHO, 2017). VAD can be addressed in part by increasing the content of particular forms of carotenoids in the edible portion of crops that are consumed in areas affected by this nutritional problem. Carotenoids including α‐, β‐ and γ‐carotenes and β‐cryptoxanthin possessing β‐ionone rings can be converted by the body into vitamin A retinol, and thus often termed provitamin A (Grune *et al*., 2010; Yeum and Russell, 2002). Retinol is a component of rhodopsin, an essential protein for light perception by the eye (Zhong *et al*., 2012). Of the different carotenoids, the all‐*trans*‐form of β‐carotene is converted to retinol in the highest proportions and, thus, is the most nutritionally efficacious provitamin A form and the preferred target of biofortification efforts (Britton *et al*., 1998; Mayne, 1996). Cassava germplasm with elevated β‐carotene content has been identified and is currently being developed using conventional breeding strategies to address VAD in SSA (Njoku *et al*., 2014).

Despite successful development of single traits such as enhanced provitamin A, multigenic strategies are required to simultaneously address the nutritional, agronomic and postharvest storage limitations of cassava in a single variety. Combining multiple trait genes by conventional breeding in cassava is difficult because of high level of heterozygosity. In addition, desired traits, such as elevated iron and zinc accumulation in the storage roots, are not present within cassava germplasm. As such, a biotechnological approach for combining or ‘stacking’ beneficial trait genes was explored as a component of the BioCassava Plus program (Sayre *et al*., 2011). A cornerstone of BioCassava Plus was the development of provitamin A‐rich cassava, not only to meet the nutritional needs of populations that depend upon this crop as a primary food source, but also to improve agronomic performance and shelf life of harvested storage roots.

A number of metabolic engineering strategies have been used to enhance carotenoid contents of diverse plant species (Giuliano, 2017). The simplest and most successful of these strategies involved transgenic expression of bacterial or plant phytoene synthase genes. The biochemical basis for this approach is that increased activity of phytoene synthase in plastids directs enhanced flux of geranylgeranyl diphosphate (GGDP) to the biosynthesis of carotenoids. Expression of phytoene synthase alone or in combination with enzymes such as the bacterial phytoene desaturases has been used to generate substantial increases in amounts of carotenoids in a number of plants including canola (Shewmaker *et al*., 1999), rice (Paine *et al*., 2005; Ye *et al*., 2000), sorghum (Che *et al*., 2016; Lipkie *et al*., 2013), tomato (Fraser *et al*., 2002; Romer *et al*., 2000), potato (Diretto *et al*., 2007), soya bean (Park *et al*., 2017; Schmidt *et al*., 2015) and banana (Paul *et al*., 2017). Additional approaches have included the expression of plastid isoprenoid pathway enzymes. For example, the gene for 1‐deoxy‐d‐xylulose 5‐phosphate synthase (DXS), which catalyses the first step in the plastid isoprenoid pathway, was reported to enhance carotenoid concentrations in potato twofold (Morris *et al*., 2006). In addition to enhancing isoprenoid and carotenoid pathway flux, a strategy for increasing total carotenoids and β‐carotene in potato involved transgenic expression of the mutant form of the *Orange* (*Or*) from carotenoid‐rich orange cauliflower to create a presumptive carotenoid storage sink (Lu *et al*., 2006).

Provitamin A‐enriched cassava was developed by the BioCassava Plus Project through transgenic approach similar to the strategy pursued by Golden Rice (Paine *et al*., 2005; Ye *et al*., 2000). The goal of the BioCassava Plus Project was to enhance provitamin A β‐carotene in storage roots of cassava to nutritionally significant levels, targeting 40 μg/g on dry weight (DW) basis as the amount needed for 100% of the vitamin A required daily by a 2‐year‐old staple cassava consumer. Three transgenic strategies were evaluated, of which co‐expression of *DXS* and *crtB*‐encoded phytoene synthase in storage root yielded the highest concentration of β‐carotene in the storage roots. Biochemically, this approach was intended to up‐regulate the total flux in the isoprenoid pathway and to enhance the flux of isoprenoids into carotenoid synthesis. We report here the successful achievement of the target level of provitamin A carotenoids in four cassava cultivars, by presenting data from multiple glasshouse experiments and confined field trials (CFTs) conducted over several years. We also report the association of increased carotenoids with the marked reduction in the onset of PPD of cassava storage roots. However, irrespective of the cultivars used, accumulation of provitamin A carotenoids in cassava storage root was accompanied by altered carbon partitioning resulting in reduced root dry matter content (DMC). These findings were also extended to potato engineered for enhanced carotenoid production. The implications of these results for the improvement of the nutritional value, production and marketing of cassava are discussed.

## Results

### Strategies for provitamin A biofortification

Three strategies were undertaken to enhance biosynthesis and accumulation of provitamin A carotenoids in cassava storage roots. These were as follows: (i) expression of a bacterial *crtB* gene for phytoene synthase under the control of the potato patatin‐type 1 promoter, (ii) co‐expression of a *crtB* gene and the Arabidopsis *1‐deoxy‐* *d* *‐xylulose‐5‐phosphate synthase* (*DXS*) gene with both transgenes under the control of separate patatin promoters and (iii) expression of the cauliflower mutant *Orange* (*Or*) gene under the control of the potato granule‐bound starch synthase promoter (Lu *et al*., 2006). For these experiments, the *crtB* and *DXS* genes were synthesized and codon‐optimized based on codon usage information from *Arabidopsis thaliana*, due to the lack of such data for cassava at the start of the project. The *crtB* gene was also linked to coding sequence of a plastid transit peptide to target the bacterial phytoene synthase to plastids, the site of carotenoid production.

The first approach was designed to direct flux in the isoprenoid pathway from the C20 geranylgeranyl‐PP (GGDP) towards carotenoid synthesis. A similar strategy was reported previously, but yielded relatively low levels of β‐carotene at 6.7 μg/g DW in field‐grown cassava storage roots (Welsch *et al*., 2010). Our second strategy was designed to not only enhance flux from GGDP into carotenoid synthesis, but also to increase total flux through the plastid isoprenoid pathway by up‐regulation of the first step of this pathway. Our third strategy was aimed at replicating the results described for potato by expression of the cauliflower mutant *Or* gene (Lu *et al*., 2006).

### Provitamin A biofortification of cassava

**Figure 1:**
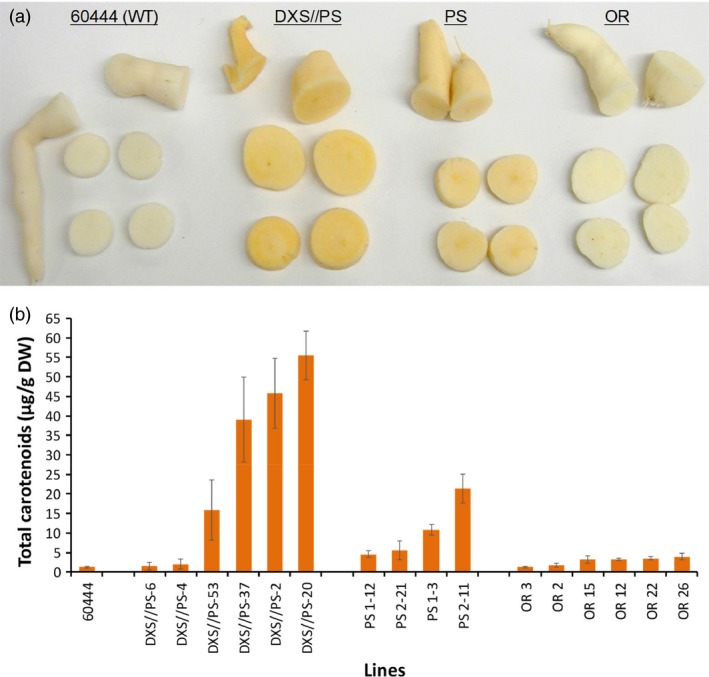
Evaluation of metabolic engineering strategies for provitamin A biofortification of cassava storage roots. (a) Sections of cassava storage roots from 12‐week‐old glasshouse‐grown nonengineered cultivar 60444 (WT) or plants engineered with transgenes for *crtB* phytoene synthase and Arabidopsis deoxy‐d‐xylulose 5‐phosphate synthase (DXS//PS), *crtB* phytoene synthase (PS) and cauliflower *Orange* gene (OR). (b) Total carotenoid concentrations of cassava storage roots from nonengineered plants or from plants engineered with the transgenes above. Roots were analysed from 12‐week‐old glasshouse‐grown plants. Bars show SE (*n* = 3–4 biological replicates).

At least 24 independent transgenic lines were recovered for each of the DXS//PS, PS (Figure S1) and Or constructs in cassava cultivar 60444. Plants PCR positive for the transgenes were grown under glasshouse conditions for 12 weeks. Storage roots were harvested and screened for the accumulation of carotenoid pigments, visibly evident as pale to deeper orange colouring. Roots visually confirmed to accumulate carotenoids were lyophilized, and carotenoid content was quantified spectrophotometrically. As shown in Figure 1, the highest levels of carotenoid accumulation were observed in lines co‐expressing *DXS* and *crtB* (DXS//PS lines). Amounts of 40–60 μg/g DW total carotenoids were detected in storage roots from the three top‐performing transgenic lines, representing a 20‐ to 30‐fold increase compared to concentrations found in storage roots harvested from nontransformed control (1–2 μg/g DW) (Figure 1). Maximum carotenoid accumulation in roots expressing *crtB* only (PS‐line) reached ~25 μg/g DW, or approximately half of the levels detected in roots co‐expressing *crtB* and *DXS*. In the case of storage roots harvested from plants transformed with the cauliflower mutant *Or* gene, low levels of carotenoid accumulation could be visually observed in roots of some of the transgenic lines. This was confirmed by spectrophotometry with total carotenoids in storage roots of the highest accumulating OR lines reaching only 3–4 μg/g DW or ~2‐fold the concentrations detected in roots of nontransgenic control (Figure 1).

HPLC analysis was conducted to further evaluate carotenoid content and compositions in the highest accumulating PS and DXS//PS lines. Quantification was achieved by the measurement of detector response for sample components relative to an internal standard (*trans*‐β‐apo‐8′‐carotenal), which agreed closely with measurements obtained spectrophotometrically for transgenic roots. Identities of the HPLC‐resolved components were established by comparisons of retention times with standards, by comparison of cassava carotenoid compositions from published results in the HarvestPlus Handbook for Carotenoid Analysis (Rodriguez‐Amaya and Kimura, 2004) and by UV absorption spectra of sample components obtained with photodiode array detection. Using this approach, carotenoids in extracts of the selected PS and DXS//PS lines were found to contain 85%–90% of all‐*trans*‐β‐carotene, the carotenoid form with the highest provitamin A activity (Figure S2). Lesser amounts of lutein, 9‐*cis*‐β‐carotene, 13‐*cis*‐β‐carotene and α‐carotene were also detected in these samples. The content of all forms of β‐carotene in extracts from storage roots of the highest accumulating DXS//PS lines was ~95% of the total carotenoids (Figure S2). HPLC analysis of extracts of additional transgenic lines indicated a general trend of a higher percentage of all‐*trans*‐β‐carotene as the total carotenoid content increased, with a maximum of ~90% all‐*trans*‐β‐carotene in the carotenoid extracts of engineered roots (Figure S2). Overall, the total content of all‐*trans*‐β‐carotene in storage roots co‐expressing *crtB* and *DXS* was 40–50 times higher than in roots from nontransgenic cassava plants, which contained 0.5–1 μg/g DW of all‐*trans*‐β‐carotene.

### Elevation of carotenoids resulted in a significant reduction in cassava storage root dry matter content

**Figure 2:**
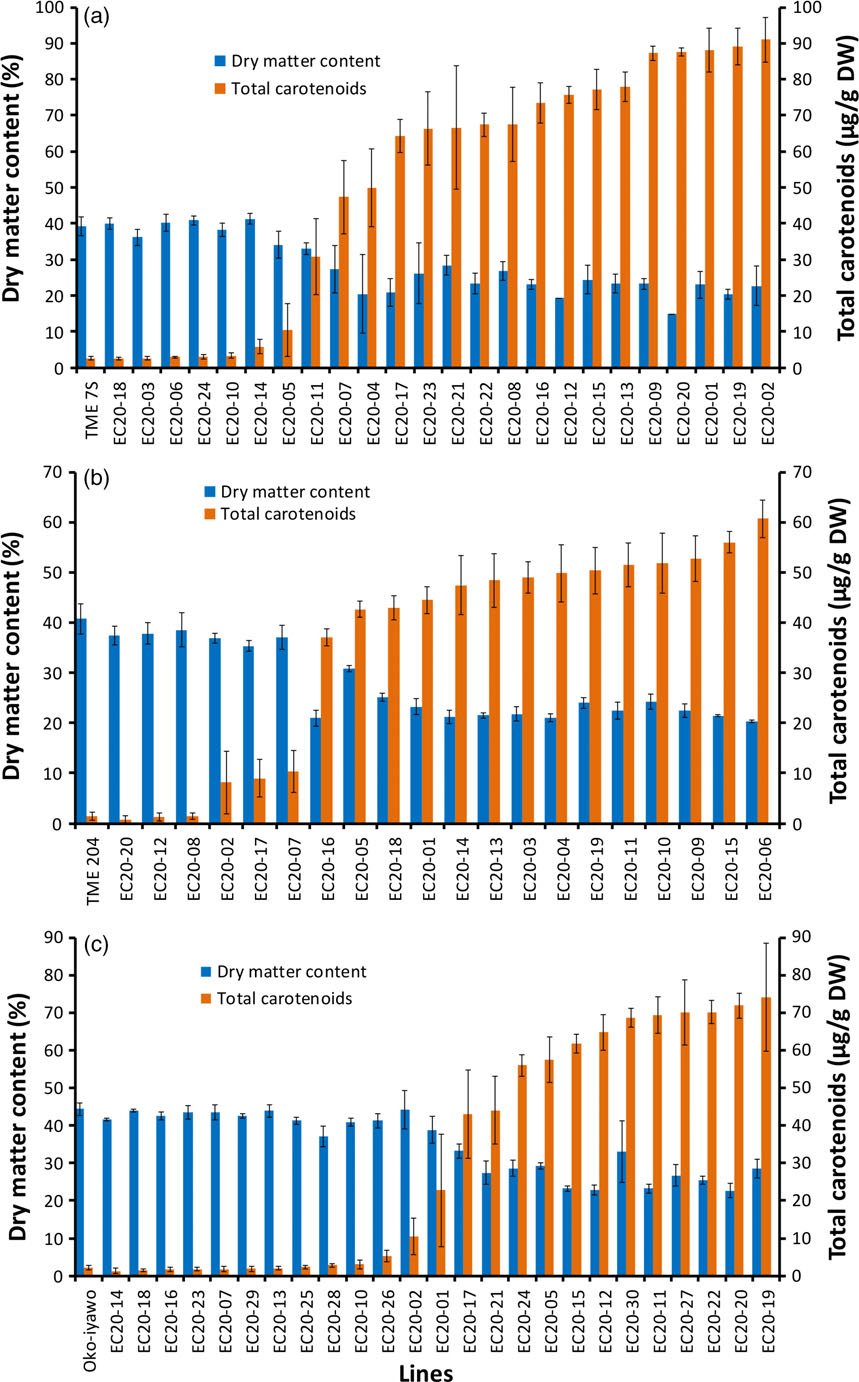
Total carotenoid and dry matter content of transgenic pEC20 lines, in cultivars (a) TME 7S, (b) TME 204 and (c) Oko‐Iyawo. Transgenic pEC20 lines co‐expressing *crtB* and *DXS* transgenes were harvested at 16–20 weeks after establishment in the glasshouse. At least three technical and four biological replicates were assayed for total carotenoid and four biological replicates for dry matter per line for each cultivar. Bars are SD.

The expression cassettes from DXS//PS in the pKAN2 binary vector were cloned into construct p5000 (Beyene *et al*., 2016a,b) and named pEC20 (Figure S1). pEC20 was used to transform three farmer‐preferred cassava cultivars TME 204, Oko‐iyawo and TME 7S (Chauhan *et al*., 2015). Between 20 and 24 independent transgenic plant lines confirmed to be co‐expressing *crtB* and *DXS* were selected per cultivar and established in the glasshouse. At harvest 16–20 weeks after planting, total carotenoid accumulation of up to 60 μg/g DW in TME 204, 70–80 μg/g in Oko‐iyawo and 80–90 μg/g DW in TME 7S was achieved. The basal levels of total carotenoids in the nontransgenic lines were as follows: 1.3 μg/g (TME 204), 2.5 μg/g (Oko‐iyawo) and 2.6 μg/g DW (TMS 7S) (Figure 2).

The DMC of storage roots in lines accumulating total carotenoids >20 μg/g DW was significantly reduced by 25%–60% in the three cultivars compared to nontransgenic controls (Figure 2). A significant inverse relationship existed between DMC and total carotenoid content for all transgenic plant lines (Figure 2a–c). To further investigate this phenomenon, over 100 transgenic TME 204 lines co‐expressing *crtB* and *DXS* were evaluated in the glasshouse and in CFT conducted at the University of Puerto Rico‐Mayaguez. Data for total carotenoids and DMC from storage roots generated in the glasshouse (*r *=* *−*0.85, P* ≤ 0.001) and the field (*r* = −0.92, *P* ≤ 0.001) showed a consistent, inverse relationship between the two traits (Figure S3). Similar findings were obtained in transgenic lines of cultivar 60444 expressing either *crtB* alone or *crtB* in combination with *DXS*, indicating that a reduction in DMC was associated with the accumulation of carotenoids and not specific to expression of *DXS* (Figure S4).

### Carotenoid accumulation in cassava storage root causes a significant change in metabolites

**Figure 3:**
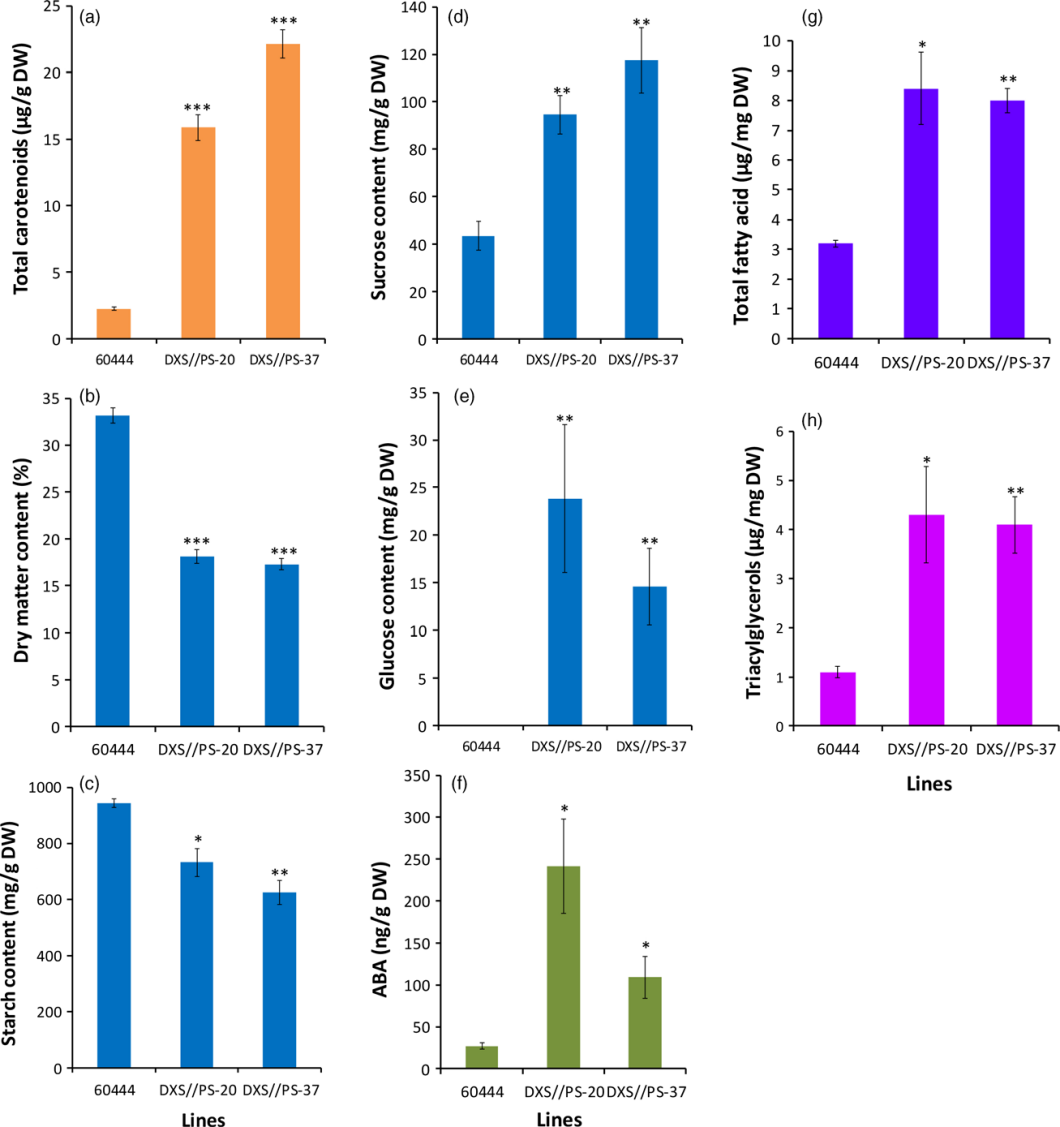
Total nonstructural carbohydrates, oil and ABA content in transgenic 60444 plant lines accumulating carotenoids. (a) Total carotenoids, (b) dry matter content, (c) starch, (d) sucrose, (e) glucose, (f) abscisic acid, (g) total fatty acid and (h) triacylglycerol content in transgenic lines DXS//PS‐20, DXS//PS‐37 and nontransgenic 60444. Cassava storage root samples from field‐grown third‐generation stake‐derived plants (SP‐3) harvested at 12 months after planting (MAP) were used for the assays. Bars are SE of three biological replicates per line; *, ** and *** stand for significant difference, respectively, at *P* ≤ 0.05, *P* ≤ 0.01 and *P* ≤ 0.001. Student's *t*‐test compared to the nontransgenic control 60444.

**Figure 4:**
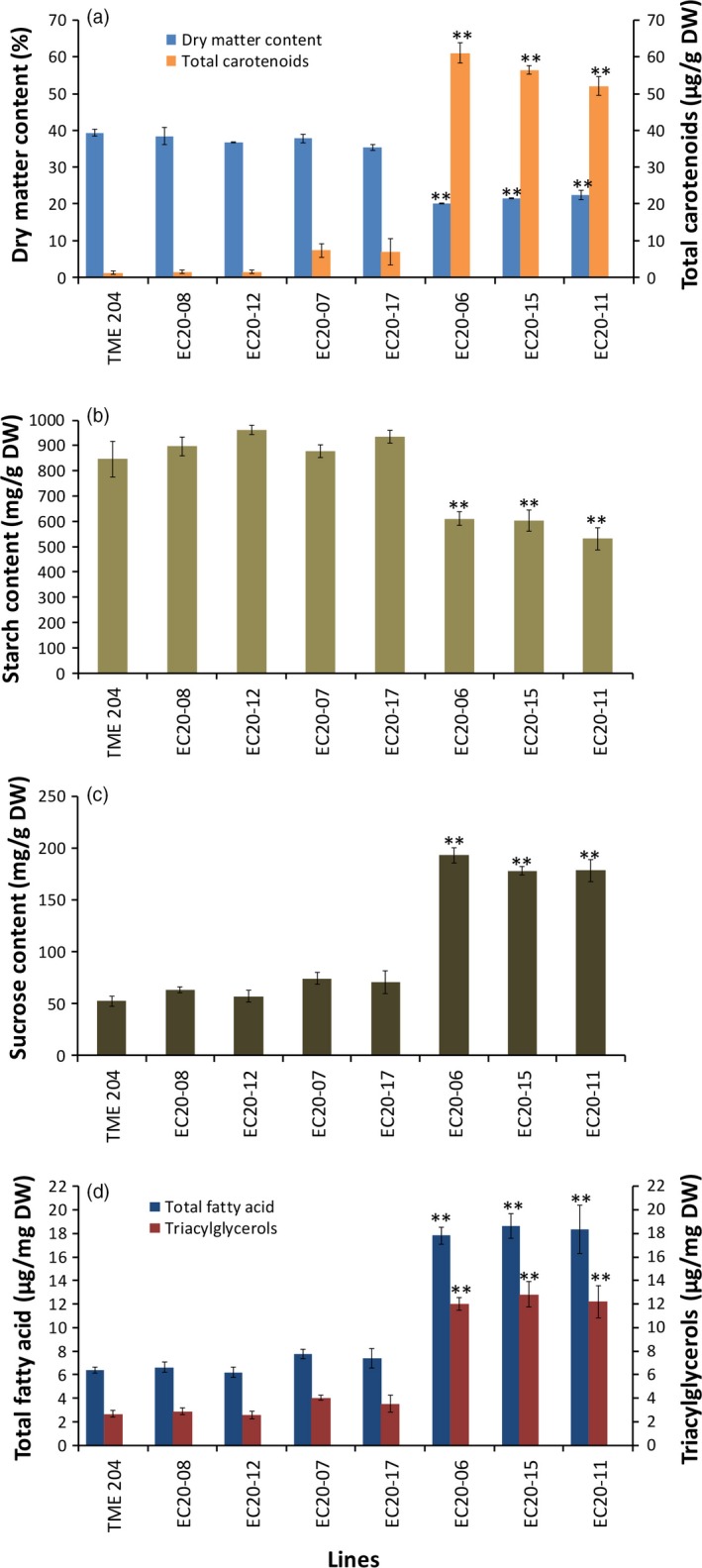
Total nonstructural carbohydrates and oil content in pEC20 transgenic TME 204 lines accumulating different levels of carotenoids. (a) Dry matter and total carotenoid, (b) starch (c) sucrose and (d) total fatty acids and triacylglycerol content. Transgenic pEC20 TME 204 lines co‐expressing *crtB* and *DXS* transgenes were grown in the glasshouse and harvested at about 16 weeks of age. Bars are SE of three biological replicates per line; ** stands for significant difference *P* ≤ 0.01. Student's *t*‐test compared to the nontransgenic TME 204.

Starch accounts for 74%–85% of cassava storage root on DW basis (Rickard *et al*., 1991). The significant DMC reduction observed in high carotenoid‐accumulating lines prompted us to assess total nonstructural carbohydrates (starch, sucrose and glucose) in transgenic cassava storage roots. Carotenoid‐accumulating transgenic DXS//PS lines from cultivar 60444 were established under CFT in Puerto Rico and storage roots harvested at 12 months after planting (MAP). These lines showed a significant reduction of 23%–34% in starch in DXS//PS‐expressing lines compared to nontransgenic 60444 grown in the same field (Figure 3c). A similar level of starch reduction (28%–37%) was observed within glasshouse‐grown storage roots of transgenic pEC20 lines of cultivar TME 204 (Figure 4b). Concomitant with reduced starch content, storage roots from these plants possessed elevated sucrose concentrations at a 2.1‐ to 3.7‐fold increase, compared to the nontransgenic controls (Figures 3d and 4c). A 14‐ to 24‐fold increase in glucose content was also seen in transgenic 60444 lines (Figure 3e). In addition to total soluble carbohydrates, total fatty acid concentrations were increased two‐ to threefold, and triacylglycerols were increased by greater than fourfold in carotenoid‐accumulating lines compared to nontransgenic controls in both 60444 and TME 204 (Figures 3g,h and 4d). These data indicate a cultivar‐independent metabolite shift from starch to soluble carbohydrates and oil in transgenic carotenoid‐accumulating storage roots (Figures 3 and 4). Given that abscisic acid (ABA) is synthesized from β‐carotene (Finkelstein, 2013), levels of this hormone were assayed to assess a possible link between ABA and β‐carotene concentrations in storage roots. Notably, transgenic cassava lines had two‐ to threefold greater levels of ABA within their storage roots than nontransgenic controls (Figure 3f).

### Elevation of carotenoids in potato tubers had similar effect as in cassava storage roots

**Figure 5:**
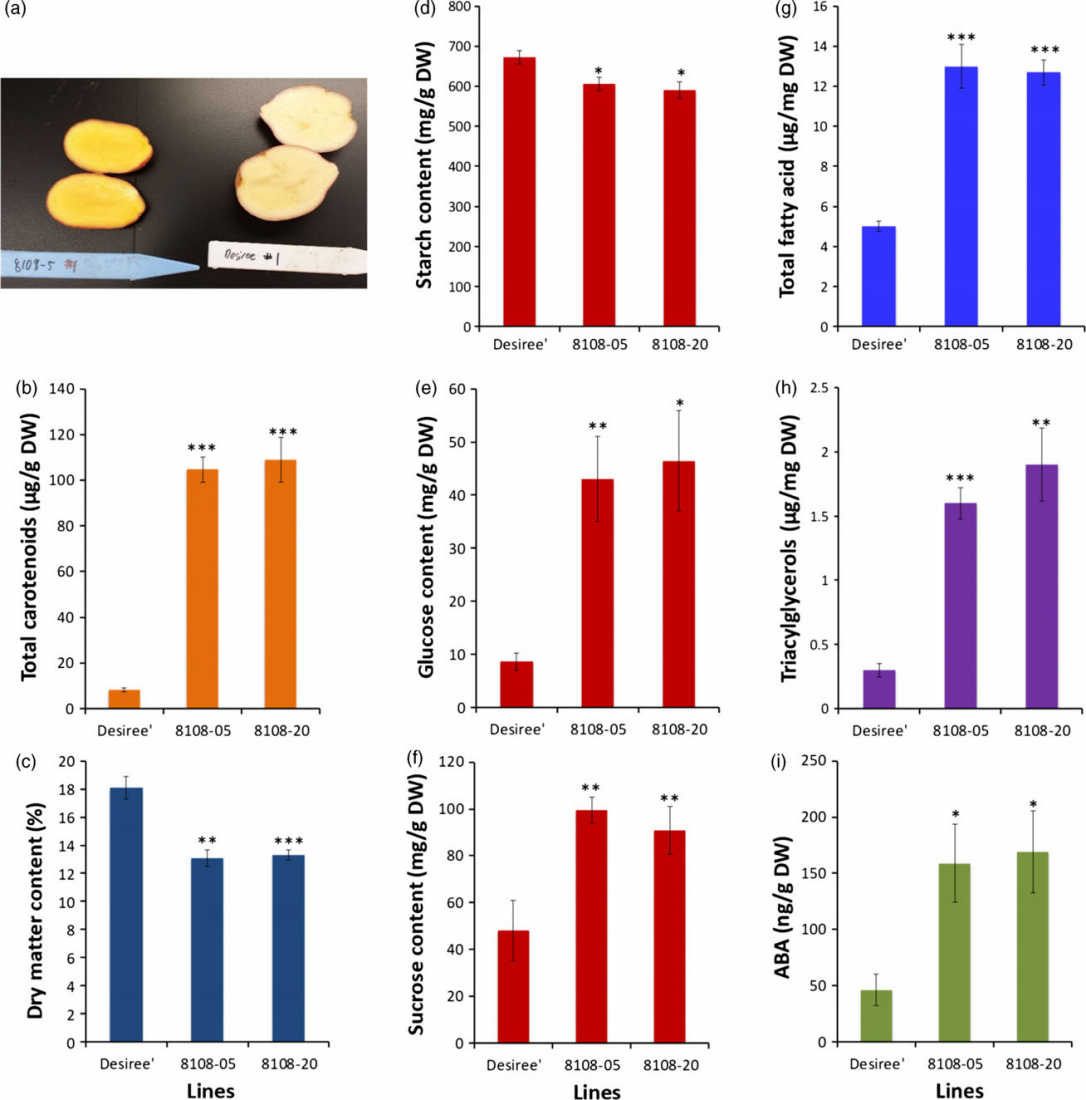
Total carotenoid accumulation and tuber metabolite in transgenic potato co‐expressing *crtB* and *DXS* transgenes. (a) Orange‐fleshed potato line 8108‐05 and nontransgenic Desiree, (b) total carotenoid, (c) dry matter, (d) starch, (e) glucose, (f) sucrose, (g) total fatty acid, (h) triacylglycerols and  (i) abscisic acid content in two transgenic potato lines 8108‐05 and 8108‐20 expressing *crtB* and *DXS* and nontransgenic Desiree. Data are average of 4–6 plants per line, and bars are SE. *, ** and *** stand for significant difference, respectively, at *P* ≤ 0.05, *P* ≤ 0.01 and *P* ≤ 0.001. Student's *t*‐test compared to the nontransgenic Desiree.

To determine whether reductions in DMC and changes in starch, sucrose and fatty acids were specific to cassava roots engineered for enhancing carotenoid concentrations, a parallel study was conducted in potato (*Solanum tuberosum*). Transgenic potato (cv. Desiree) lines were generated expressing the same *crtB* and *DXS* transgenes used in cassava (Figure S1). Ten plants confirmed to be positive for the presence of the selectable marker gene were grown in the glasshouse for 14 weeks. Upon harvest, carotenoid concentrations in tubers from eight independent lines ranged from 37 to 109 μg/g DW as compared to about 8 μg/g DW in the nontransgenic cultivar Desiree (Figure 5a,b; Figure S5a). Similar to cassava, an inverse relationship was observed between total carotenoids and DMC (Figure 5c, Figure S5b). Of the eight transgenic lines, potato tubers from the two high carotenoid‐accumulating lines 8108‐5 and 8108‐20 with 104 μg/g DW and 109 μg/g DW total carotenoids, respectively, and the nontransgenic Desiree (Figure 5a,b) were analysed for starch, sucrose, glucose and fatty acid content. DMC of tubers from these lines was reduced by ~25% compared to tubers from nontransgenic controls. Similar to cassava, starch concentrations of tubers from high carotenoid lines decreased, while sucrose, glucose, total fatty acid, triacylglycerols and ABA levels were significantly higher than nontransgenic controls (Figure 5c–i). These data clearly demonstrated that elevation of carotenoids in potato tubers is accompanied by metabolic changes similar or nearly equivalent to those observed in cassava.

### Provitamin A biofortification prolongs shelf life of cassava storage roots

**Figure 6:**
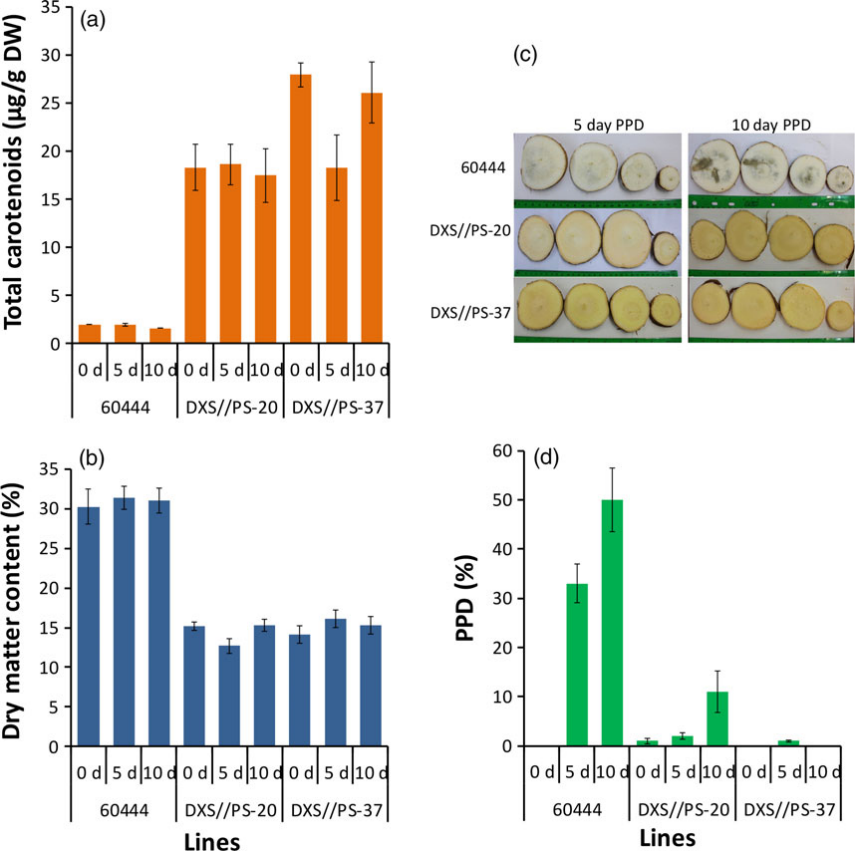
Effects of carotenoid accumulation on postharvest physiological deterioration (PPD). (a) Total carotenoids, (b) dry matter content, (c) slices of storage roots of nontransgenic 60444 (top row) and the two transgenic lines (middle and bottom rows) and (d) PPD of storage roots. Storage roots were harvested from second‐generation stake‐derived plants at 12 months after planting (MAP) from confined field trial conducted in Puerto Rico. Transgenic cassava lines with increased carotenoids (DXS//PS‐20 and DXS//PS‐37) and the nontransgenic 60444 were used. Roots were assessed after storage in a dry, ventilated area away from direct sunlight for 0, 5 and 10 days after harvest. Sections of roots were cut transversely at 20%, 40%, 60% and 80% from the proximal to distal ends (left to right). Day 0 is not shown due to the lack of PPD. Bars shown are SE for three biological replicates.

Postharvest physiological deterioration in cassava causes a significant loss of storage roots, reducing the food, feed and market value of the crop. The impact of carotenoid accumulation on PPD in cassava storage roots was evaluated in the two transgenic 60444 lines (DXS//PS‐20 and DXS//PS‐37) from plants grown under CFT conditions in Puerto Rico. At 12 MAP, total carotenoid concentrations of storage roots harvested from these plants were 18.1 and 24.1 μg/g DW, respectively, compared to 1.8 μg/g DW in the nontransgenic 60444 (Figure 6) with the DMC reduced to 14.4% and 15.7%, respectively, in contrast to 30.9% in control. Visual assessment of PPD at 5 and 10 days after harvest showed nontransgenic roots with 33% and 50% deterioration at these time points, respectively, but over the same time periods, storage roots harvested from transgenic line DXS//PS‐20 had PPD of only 2% and 11%, and line DXS//PS‐37 1% and 0% PPD, respectively (Figure 6c). No significant change in carotenoid and DMC was observed at 5 and 10 days of storage (Figure 6).

### Expression of starch biosynthetic genes is reduced, while genes involved in ABA biosynthesis are up‐regulated in storage roots of carotenoid‐accumulating lines

**Figure 7:**
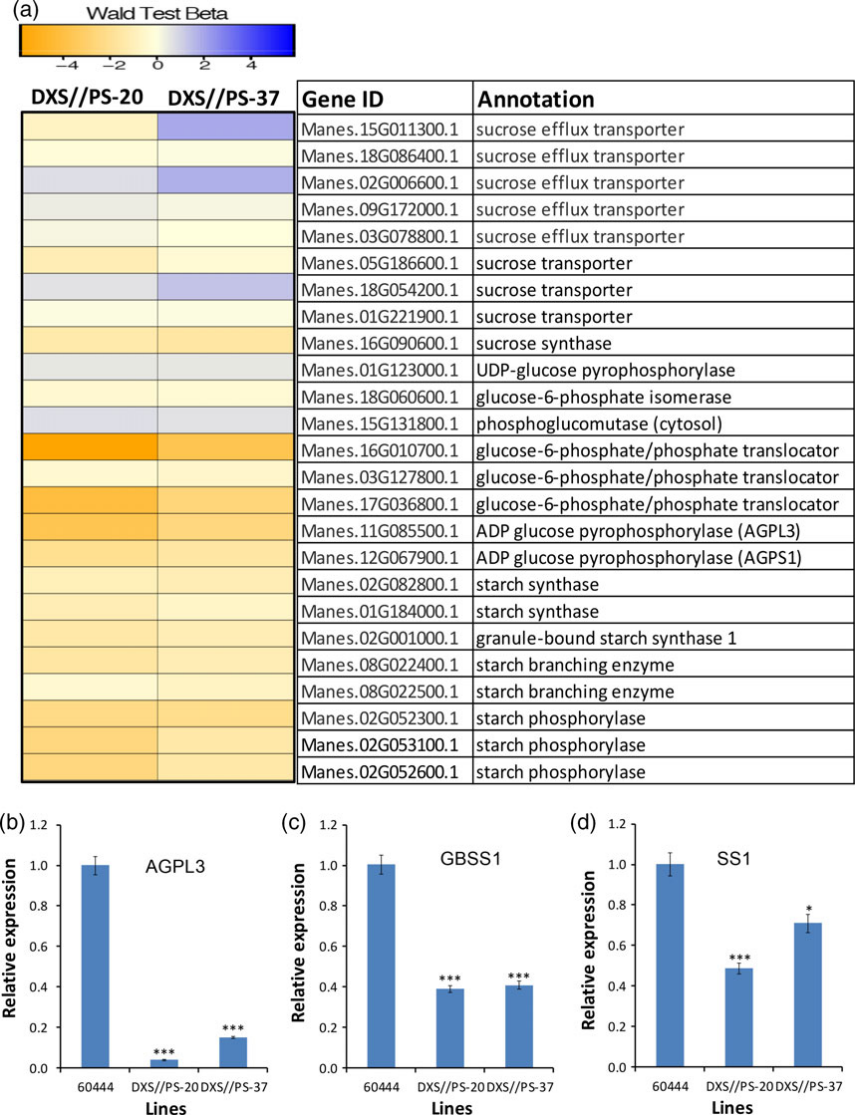
Effect of carotenoid accumulation on expression of genes in starch biosynthetic pathway in cassava storage roots. (a) Heatmap showing differentially expressed genes involved in sugar transport and starch biosynthesis in the storage roots of two transgenic cassava lines (DXS//PS‐20 and DXS//PS‐37) compared to nontransgenic 60444, (b) RT‐qPCR validation of cassava ADP‐glucose pyrophosphorylase 3 (MeAGPL3, Manes.11G085500.1), (c) RT‐qPCR validation of cassava granule‐bound starch synthase I (MeGBSSI, Manes.02G001000.1) and (d) RT‐qPCR validation of cassava soluble starch synthase I gene (MeSSI, Manes.01G184000.1) in transgenic lines accumulating carotenoids and nontransgenic 60444. Genes presented were selected based on Wang *et al*. (2014) from RNA‐seq data generated from third‐generation stake‐derived field‐grown plants (SP‐3) harvested at 12 MAP. Total carotenoid, dry matter content and other metabolite data generated from these lines are presented in Figure 3. *, *** stand for significant difference, respectively, at *P* ≤ 0.05 and *P* ≤ 0.001. Student's *t*‐test compared to nontransgenic 60444.

Transcriptome was assessed to identify differentially expressed genes in storage roots of carotenoid‐accumulating cassava lines DXS//PS‐20 and DXS//PS‐37 and nontransgenic 60444 controls harvested from the field at 12 MAP. Approximately 8000 and 9000 genes in DXS//PS‐20 and DXS//PS‐37, respectively, were found to be differentially expressed compared with the nontransgenic control. Of these, ~6000 genes were common to both transgenic lines. Of interest among the differentially expressed genes were those involved in starch biosynthesis (Figure 7a). Genes encoding sucrose synthase, glucose‐6‐phosphate/phosphate translocator, ADP‐glucose pyrophosphorylase large (Manes.11G085500.1) and small subunits (Manes.12G067900.1), starch synthase, starch‐branching enzymes and starch phosphorylases were significantly down‐regulated in roots from transgenic, carotenoid‐accumulating lines compared to roots from the nontransgenic control (Figure 7a). The RNA‐seq data were further validated by qPCR analysis of selected genes: ADP‐glucose pyrophosphorylase (MeAGPL3, Manes.11G085500.1), granule‐bound starch synthase 1 (GBSS1, Manes.02G001000.1) and soluble starch synthase (Manes.01G184000.1). Consistent with transcriptome data, these genes showed a significant reduction in expression in DXS//PS‐20 and DXS//PS‐37 compared with the nontransgenic control (Figure 7b,c,d). Consistent with the two‐ to fourfold elevation in ABA concentrations in transgenic cassava storage roots (Figure 3f) and potato tubers (Figure 5i), genes involved in ABA biosynthesis were significantly elevated, including genes encoding β‐carotene hydroxylase (MeBCH1, Manes.06G152200.1) that converts β‐carotene to zeaxanthin, *9‐cis*‐epoxycarotenoid dioxygenase (NCED3, Manes.15G102000.1 and Manes.15G050500.1) and short‐chain dehydrogenase/reductase (ABA2, Manes.07G052100.1, Manes.11G077300.1) in the engineered carotenoid‐accumulating roots relative to nontransgenic roots. Despite the increases in total fatty acids and triacylglycerols associated with carotenoid accumulation, little or no up‐regulation was detected in the expression of genes for selected enzymes involved in directing carbon flux into fatty acid biosynthesis (pyruvate dehydrogenase and acetyl‐CoA carboxylase subunits), *de novo* fatty acid biosynthesis (β‐ketoacyl‐acyl carrier protein synthase I and III) or fatty acid storage (diacylglycerol acyltransferase 1, 2 and 3) (Table S2).

## Discussion

We report the enhanced accumulation of provitamin A β‐carotene in cassava storage roots of field‐grown plants to concentrations at least as high as those in seeds of Golden Rice 2 (Paine *et al*., 2005) by co‐expression of transgenes for a bacterial phytoene synthase (*crtB*) and Arabidopsis 1‐deoxy‐d‐xylulose 5‐phosphate synthase (*DXS*). Targeted levels of 40 μg/g DW were demonstrated in farmer‐preferred cultivars. Accumulation of carotenoids was accompanied by marked reductions in PPD of storage roots, a process that limits the full potential of cassava as a food, feed and industrial crop. An unexpected consequence of our metabolic engineering strategy was reduced DMC of cassava storage roots, arising primarily from reduced starch content and compensatory increases in concentrations of soluble sugars and triacylglycerols. We show that this phenomenon can be replicated in potato engineered for enhanced carotenoid accumulation in tubers, suggesting that altered carbon partitioning is not specific to cassava, but a more general outcome of carotenoid biofortification of starch‐rich root and tuber crops. Finally, comparative evaluation of transcriptomes of storage roots from field‐grown engineered and nonengineered plants revealed possible targets for restoring starch accumulation to levels found in roots from nonengineered plants and for obtaining additional enhancements of β‐carotene accumulation.

Vitamin A deficiency is a pervasive major health issue affecting an estimated 47% of preschool children in SSA (FAO, 2017). This deficiency results in increased incidence of blindness and suppressed immunity leading to enhanced mortality (Mayne, 1996). While cassava is a major caloric source in SSA, it is a relatively poor source of micronutrients, including provitamin A β‐carotene (Sayre *et al*., 2011). Development of cassava storage roots with increased β‐carotene through breeding or biotechnology, as described here, offers a viable solution to address a major nutritional challenge in SSA. Processing methods used to make popular cassava food products such as gari and fufu have been shown to reduce β‐carotene concentrations of biofortified cassava by 30% for gari preparation and 65% for fufu preparation (Failla *et al*., 2012). Based on the accumulation of all‐*trans*‐β‐carotene to concentrations of ≥35 μg/g DW in engineered storage roots and a conservative estimate of 12 : 1 for conversion of all‐*trans*‐β‐carotene to retinol (vitamin A) by the body (Van Loo‐Bouwman *et al*., 2014), ~160 g to ~211 g DW of our cassava storage root powder prepared for fufu would need to be consumed daily to obtain the vitamin A RDA for preschool children (300–400 μg retinol activity equivalents) or approximately 20‐fold more than can be obtained from nonengineered cassava storage roots. Of note, a conversion factor of all‐*trans*‐β‐carotene to retinol as high as 3.7 : 1 (on a weight basis) has been demonstrated in model animal studies (Howe *et al*., 2009), suggesting that lower amounts of biofortified cassava may be sufficient to attain RDA levels.

A major limitation for cassava utilization as a food crop is the short shelf life of harvested storage roots (Djabou *et al*., 2017; Reilly *et al*., 2004). Within approximately 2 days following the removal of cassava storage roots from plants, PPD onset can be observed by the initiation of dark coloration in the root vascular parenchyma (Beeching *et al*., 2002). PPD eventually results in a complete dark ring encompassing the root vasculature, as shown in Figure 6c for nonengineered roots stored for 10 days. PPD, which is controlled in part by reactive oxygen species (ROS) accumulation, results in the need to process roots immediately following harvest for consumption. This constrains the ability to transport harvested cassava storage roots from rural production areas to urban markets (Sayre *et al*., 2011). Not only does this impact the use of cassava for food production, but it also limits cassava's potential as a cash crop for farmers (Sayre *et al*., 2011). A positive correlation between the delay of PPD onset and carotenoid concentrations of yellow flesh varieties of cassava has been previously reported (Sánchez *et al*., 2006). Our findings, although difficult to compare directly with this previous report, are consistent. In the current study, a transgenic line with the highest levels of carotenoids displayed no detectable PPD after 10 days of storage. At this time point, PPD had reached a level of ~50% in our scoring system in roots of nonengineered plants. While we cannot exclude the possibility that this extreme delay of PPD is due to a decreased DMC of roots, a more likely explanation is that the accumulation of carotenoids results in enhanced antioxidant capacity to suppress ROS accumulation in the engineered roots, as has been shown in roots engineered for enhanced alternative oxidase expression (Zidenga *et al*., 2012). Nevertheless, the exact mechanism of delayed PPD associated with carotenoid accumulation requires additional experimentation.

Our biotechnological approaches to enhance the provitamin A content of cassava storage roots complement ongoing selection efforts to identify naturally occurring varieties with roots enriched in carotenoids (Carvalho *et al*., 2016; Njoku *et al*., 2014; Welsch *et al*., 2010). The advantages of biotechnological approaches include the potential to transfer the metabolic engineering strategy to any cassava cultivar, including farmer‐preferred varieties that are well adapted to diverse regions of the SSA, and the ability to combine or ‘stack’ provitamin A with other traits that are difficult to achieve through conventional breeding, such as virus resistance and iron biofortification. Notably, the combination of *crtB* and *DXS* transgene co‐expression resulted in β‐carotene concentrations that were approximately twofold higher than *crtB* expression alone in our experiments and approximately fivefold higher than reported for *crtB* expression with the cassava *CP1* promoter (Welsch *et al*., 2010). In the latter case, the difference in carotenoid accumulation between the two‐transgene combination (*crtB*,* DXS*) and *crtB* expression alone may be due in part to the use of different promoters.

Despite the demonstration of the efficacy of our strategy, the engineered cassava roots had significant reductions in DMC (Figure 2) largely due to lower starch content than those in nonengineered roots and partially compensating increases in soluble sugars and oil (triacylglycerols) (Figures 3 and 4). To our knowledge, this phenotype has not been previously reported in other starchy plant organs engineered for enhanced carotenoid concentrations. To examine whether this phenotype is limited to cassava, we introduced the *crtB*//*DXS* transgene combination into potato. Similar to cassava, the engineered potato tubers had large increases in carotenoid concentrations accompanied by a reduction in DMC, starch and increased soluble sugars and triacylglycerols (Figure 5). ABA concentrations were also elevated in cassava storage roots and potato tubers, likely arising from the conversion of β‐carotene via catabolic enzymes such as β‐carotene hydroxylase. These findings suggest that reduced DMC may be a more general phenomenon in provitamin A‐biofortified starch crops. Similar to the transgenic cassava reported here, conventionally bred yellow‐fleshed cassava also showed reduced DMC (Esuma *et al*., 2016a,b; Njoku *et al*., 2015). Ceballos *et al*. (2013), however, reported that multigenerational selection for combined high DMC and high carotenoid content may minimize negative effects of carotenoid accumulation on DMC. A similar inverse relationship between dry matter and carotenoid content has also been reported in orange‐fleshed sweet potato developed through conventional breeding (Vimala *et al*., 2011).

Transcriptomic studies of cassava storage roots conducted to understand the metabolic basis for these phenotypes revealed reductions in the expression of genes encoding ADP‐glucose pyrophosphorylase, starch synthase and starch pyrophosphorylase in engineered storage roots versus nonengineered roots. Conversely, expression of genes for selected sucrose efflux transporters and sucrose transporters was up‐regulated in the engineered roots compared to nontransgenic roots. Given the importance of ADP‐glucose pyrophosphorylase for the regulation of carbon partitioning between starch biosynthesis and sucrose and fatty acid biosynthesis (Ballicora *et al*., 2003), it is likely that down‐regulation of genes (Manes.11G085500.1; Manes.12G067900.1) for this enzyme accounts for the shift in metabolic flux observed in the engineered roots. Although not reported to our knowledge for the plant counterpart, the *E. coli* ADP‐glucose pyrophosphorylase is allosterically activated by pyruvate (Ballicora *et al*., 2003). It is possible that diversion of pyruvate for carotenoid synthesis via the plastidic methylerythritol 4‐phosphate pathway may suppress ADP‐glucose pyrophosphorylase activity by reduced expression of the corresponding gene. While a relatively small amount of pyruvate may be consumed for carotenoid biosynthesis compared to the overall concentration of starch in roots, our transcriptomic studies and evidence for ABA accumulation suggest that carotenoid catabolic pathways are up‐regulated in the engineered roots. In the absence of metabolic flux data, it is not clear how much pyruvate is ultimately used by enhanced carotenoid production and associated breakdown. In cereals, increased ABA levels are directly correlated with increased seed size and starch content (Bai *et al*., 1989; Kato *et al*., 1993; Seiler *et al*., 2011). In developing maize endosperm, the effect of ABA is mediated by the ABA‐insensitive 4 (ABI4) transcription factor that binds to the *cis*‐regulatory element ‘CACCG’ and activates sucrose synthase I gene expression (Hu *et al*., 2012). As presented in our study, elevated ABA is negatively correlated with starch and DMC in cassava storage roots and potato tubers, indicating possible differences between these storage organs and seeds. These findings highlight the need for greater understanding of the metabolic links between starch and carotenoid biosynthesis in biofortification studies of starch crops, such as cassava, potato and sweet potato, and more importantly the molecular mechanism that triggers this shift in metabolism. It is notable that phenotypes that we observed in carotenoid‐enriched cassava and potato, including enhanced triacylglycerol and ABA accumulation, were opposite of phenotypes observed in soya beans engineered for β‐carotene production (Schmidt *et al*., 2015), suggesting that different metabolic perturbations are associated with carotenoid enhancement in starch‐ and oil‐rich storage organs.

Overall, our findings demonstrate the efficacy of *crtB//DXS* transgene expression for obtaining nutritionally significant concentrations of provitamin A and suppressing PPD in cassava storage roots. Our results also point to additional potential targets improving the effectiveness of our metabolic engineering strategy. These include enhancing ADP‐glucose pyrophosphorylase gene expression to restore starch accumulation to concentrations comparable to those in nontransgenic storage roots and reducing expression of the gene for β‐carotene hydroxylase, which controls the first step in β‐carotene catabolism.

## Experimental procedures

### Constructs, transgenic plant production and characterization under glasshouse/field conditions

Details of gene expression vector construction and production and establishment of transgenic plants in the glasshouse and field are provided in Supporting Information. Briefly, the *crtB* gene from *Pantoea ananatis* and *DXS* gene from *A. thaliana* were codon‐optimized based on *A. thaliana* codon usage data and commercially synthesized (GenBank Accession numbers JN374901, JN374902). A plastid transit peptide sequence from the coriander ∆4 acyl‐ACP desaturase cDNA (Cahoon and Shanklin, 2000) was added at the 5′‐end of the synthesized *crtB* gene. The subsequent *crtB* and *DXS* genes were cloned under the control of the promoter for *S. tuberosum* patatin‐type 1 gene, and constructs containing the *crtB* expression cassette alone or in combination with the *DXS* cassette were introduced into friable embryogenic callus produced from cassava cultivars 60444, TME 7S, TME 204 and Oko‐iyawo (Chauhan *et al*., 2015; Taylor *et al*., 2012).

### Determination of PPD in cassava storage roots

Harvested storage roots were stored at ambient temperature in a well‐ventilated and dry area protected from direct sunlight. PPD was assessed on the storage root at 0, 5 and 10 days after harvest. Storage root was cut at 20%, 40%, 60% and 80% from the proximal to distal end with each slice being subjected to total carotenoids, dry matter and PPD analysis. PPD was assessed visually by assigning a score 0%–100% as described in Salcedo *et al*. (2010). Data from slices per root were averaged for final analysis.

### Dry matter and carotenoid measurement

Harvested storage roots were cleaned of the soil and sliced transversely into sections along the length of the root. The peel was removed and root sections were cleaned by washing under running tap water and blotted dry using paper towels. Samples were immediately placed in 50‐mL conical tubes; fresh weight was recorded and samples were frozen in liquid nitrogen. Samples were lyophilized for ≥48 h with tubes, and lyophilizer jars were wrapped in aluminium foil to protect from light and subsequently weighed to determine DMC. Samples were ground to flour using FastPrep‐24 (MP Biomedical, Solon, OH) in 50‐mL conical tubes using ceramic beads and immediately used for analysis or stored at −80 °C until needed.

For spectrophotometric analysis of total carotenoids, 25–60 mg of lyophilized cassava flour was extracted in 1.5 mL of diethyl ether in a 13 × 100 mm capped glass tube with agitation for 60 min in the dark on a nutating mixer. Following centrifugation, the extract was analysed by spectrophotometry at 641.8, 660 and 470 nm and carotenoid concentration calculated using the formula described by Lichtenthaler (1987). HPLC analysis of carotenoid content was performed on 100–150 mg lyophilized cassava flour obtained from carotenoid‐biofortified roots or ~300 mg from nonengineered roots. Carotenoids were extracted in 1.5 mL of acetone as described above with 500 ng of *trans*‐β‐apo‐8′‐carotenal (Sigma, Saint Louis, MO) added as an internal standard (Shewmaker *et al*., 1999). HPLC conditions used for carotenoid analyses were essentially as described (Rodriguez‐Amaya and Kimura, 2004) with details provided in Supporting Information. At least three biological replicates were conducted for spectrophotometric and HPLC analyses.

### Total fatty acid and triacylglycerol measurement

Total fatty acids and triacylglycerols were extracted, analysed and quantified similar to the previously described protocol (Msanne *et al*., 2012; Zhu *et al*., 2016), except that triacylglycerols were purified by silica solid‐phase extraction rather than by thin‐layer chromatography. Detailed methodology is provided in the Supporting Information.

### Determination of nonstructural carbohydrates and ABA

Total nonstructural carbohydrates (glucose, sucrose, starch) were evaluated from ~100 mg of storage root samples. The method used was modified from those described in Cairns (1987), Rasmussen and Henry (1990), Hendrix (1993) and Gomez *et al*. (2007). Soluble sugars (glucose and sucrose) were extracted with 80% (v/v) ethanol by incubation in 80 °C water bath for 60 min, with occasional vortexing. Tubes were then centrifuged at 14 000 ***g*** for 5 min and supernatant was collected. Pellets in each tube were further extracted twice, and all extracts were pooled. Aliquots of extracts were incubated in an invertase solution (EC 3.2.1.26; Sigma, Saint Louis, MO) for 4 h at room temperature to hydrolyse the sucrose. The ethanol‐extracted residue was washed, suspended in 500 μL of water and placed in a boiling water bath for 30 min to gelatinize the starch, followed by incubation at 55 °C for ≥2 h in a 0.2 M NaOAc (pH 5.1) solution containing amyloglucosidase (EC 3.2.1.3; Sigma) and α‐amylase (EC 3.2.1.1; Sigma) to hydrolyse the starch. Glucose, sucrose and starch were assayed as glucose equivalents following the glucose oxidase (EC 1.1.3.4), peroxidase (EC 1.11.1.7) enzymatic technique in 96‐well microplates (Gomez *et al*., 2007; Hendrix, 1993). For ABA determination, 50 mg of freeze‐dried and ground cassava storage root or potato tuber was used following an established mass spectrometry‐based protocol (Pan *et al*., 2010).

### Real‐time quantitative PCR

Total RNA was extracted from ~50 mg of freeze‐dried, ground flour of cassava storage root samples using the cetyltrimethylammonium bromide protocol (Doyle and Doyle, 1990) and genomic DNA was removed using the TURBO DNA‐free Kit (Ambion, Carlsbad, CA). Synthesis of cDNA and Real‐time quantitative PCR (RT‐qPCR) analysis were performed as described by Ogwok *et al*. (2015). At least three biological and two technical replicates were run per sample. Primers used and the respective cassava genes analysed are presented in Table S1. Cassava serine/threonine‐protein phosphatase 2A was used as housekeeping gene for the normalization of expression values (Moreno *et al*., 2011).

### Transcriptome analysis

Transcript analysis was performed on the two transgenic 60444 lines DXS//PS‐20 and DXS//PS‐37 and the nontransgenic control. The transgenic lines were obtained from third‐generation stake cuttings (SP‐3) grown in the field in Puerto Rico. Samples of storage roots were collected at 12 MAP from three biological replicates per line. Upon harvest, roots were peeled, frozen immediately in liquid N_2_, freeze‐dried and shipped to University of Nebraska for total RNA extraction as described by Kumar *et al*. (2007). For library preparation, an Illumina TruSeq sample preparation kit with polyA mRNA selection was used with 1 microgram of total RNA per sample, following the manufacturer's instructions (Illumina). Nine libraries were pooled and sequenced using an Illumina HiSeq 2000 with paired‐end reads of 101 bp at MOgene LC. HiSeq data was quality‐trimmed, filtered and paired using Trimmomatic version 0.36 (Bolger *et al*., 2014) with parameters phred = 33, leading = 3, trailing = 3, slidingwindow = 4 : 15, minlen = 36. A total of 155 974 024 reads were obtained and reduced to 145 752 673 reads with an average of 29 150 535 reads per sample and a standard deviation of 1 704 123 reads following quality trimming and paring. Kalisto version 0.42.4 (Bray *et al*., 2016) was used to index transcripts with the *M. esculenta* transcript file [Mesculenta_305_v6.1.transcript.fa.gz; Phytozome; (Bredeson *et al*., 2016)] using default parameters. Differential expression between transgenic and nontransgenic lines was quantified using Sleuth (Pimentel *et al*., 2017). The Wald test (Chen *et al*., 2011) ß is reported, which is a bias estimator, and approximately the log‐fold change in gene expression over the control sample. Raw and processed data are available via the Gene Expression Omnibus at GSE100319 (https://www.ncbi.nlm.nih.gov/geo/query/acc.cgi?acc=GSE100319).

## Conflict of interest

The authors have no conflict of interest to declare.

## Author contributions

EC, NT, RTS and PA conceived the project. GB, FRS, RDC, DS, NN, EG, JG, RLS, MF and JJ designed the provitamin A cassava experiments and generated data. GB, MG, IM and EC analysed experimental data. JVE and EL generated and characterized transgenic provitamin A potato. GB and EC wrote the manuscript. All authors reviewed and commented on the manuscript.

## Supporting information


**Figure S1** Maps of the T‐DNA regions of the different constructs used for provitamin A enhancement of cassava storage roots.
**Figure S2** Representative chromatogram from the HPLC analysis of storage roots from DXS//PS engineered plants.
**Figure S3** Dry matter and total carotenoid content of transgenic p8001 TME 204 cassava lines.
**Figure S4** Dry matter and total carotenoid content of transgenic lines expressing *crtB* alone (PS‐lines) or co‐expressing crtB and DXS (DXS//PS‐lines).
**Figure S5** Total carotenoid and dry matter content in transgenic p8108 potato lines co‐expressing *crtB* and *DXS*.
**Table S1** List of genes and primer pairs used for cloning, detection and expression of the transgenes and expression analysis of cassava starch biosynthetic genes.
**Table S2** Selected differentially expressed genes associated with generation of carbon precursors for fatty acid biosynthesis (pyruvate dehydrogenase, acetyl‐CoA carboxylase), *de novo* fatty acid biosynthesis (β‐ketoacyl acyl carrier protein synthase I, III), or fatty acid storage as triacylglycerols (diacylglycerol acyltransferases 1, 2, 3) between provitamin A accumulating lines (DXS//PS‐20 and DXS//PS‐37) and wild‐type controls of cassava storage roots at 12 months after planting.
