# Supplementary material for: Provitamin A biofortification of cassava enhances shelf life but reduces dry matter content of storage roots due to altered carbon partitioning into starch
Source: Plant Biotechnol J. 2017 Dec 27;16(6):1186–200. doi: 10.1111/pbi.12862 (PMC5978869; doi:10.1111/pbi.12862)
Supplement: Supplementary file 1 — Figure S1 Maps of the T‐DNA regions of the different constructs used for provitamin A enhancement of cassava storage roots. Figure S2 Representative chromatogram from the HPLC analysis of storage roots from DXS//PS engineered plants. Figure S3 Dry matter and total carotenoid content of transgenic p8001 TME 204 cassava lines. Figure S4 Dry matter and total carotenoid content of transgenic lines expressing crtB alone (PS‐lines) or co‐expressing crtB and DXS (DXS//PS‐lines). Figure S5 Total carotenoid and dry matter content in transgenic p8108 potato lines co‐expressing crtB and DXS. Table S1 List of genes and primer pairs used for cloning, detection and expression of the transgenes and expression analysis of cassava starch biosynthetic genes. Table S2 Selected differentially expressed genes associated with generation of carbon precursors for fatty acid biosynthesis (pyruvate dehydrogenase, acetyl‐CoA carboxylase), de novo fatty acid biosynthesis (β‐ketoacyl acyl carrier protein synthase I, III), or fatty acid storage as triacylglycerols (diacylglycerol acyltransferases 1, 2, 3) between provitamin A accumulating lines (DXS//PS‐20 and DXS//PS‐37) and wild‐type controls of cassava storage roots at 12 months after planting. [file PBI-16-1186-s001.pdf]

Supporting Information

Gene expression constructs

The *crtB* and *DXS* genes used for provitamin A carotenoid overexpression were synthesized commercially. The *crtB* gene was prepared by MCLAB, San Francisco, CA based on the amino acid sequence of phytoene synthase (PS) from *Pantoea ananatis* (formerly known as *Erwinia uredovora*). The *crtB* gene sequence was codon optimized based on codon usage data of *Arabidopsis thaliana* and is available at GenBank (Accession number JN374901). The synthetic gene contained an in-frame *Bsp*HI restriction site on its 5'-end and an *Xba* I restriction site on its 3'-end and was cloned into pCR2.1-TOPO (Invitrogen, Casblad, USA). A plastid transit peptide sequence from coriander Δ4 acyl-ACP desaturase was added by ligating the *crtB* coding sequence as a *Bsp* HI/*Xba* I fragment in place of the mature Δ4 acyl-ACP desaturase coding sequence into the *Nco* I/*Xba* I sites of this clone in pBluescript SK(+) as previously described (Cahoon and Shanklin, 2000). The *DXS* coding sequence was synthesized by Celtek (Nashville, TN) based on the amino acid sequence of the Arabidopsis DXS polypeptide and was cloned into vector pUC57 with flanking 5'-*Eco* RI and 3'-*Bam* HI restriction sites. The sequence of the synthetic *DXS* gene has been deposited in GenBank (Accession # JN374902).

The *crtB* gene with added plastid transit peptide sequence and the synthetic Arabidopsis DXS were cloned downstream of the promoter for the potato (*Solanum tuberosum*) patatin-type I gene and upstream of the 3'UTR of the nopaline synthase (nos) gene from *Agrobacterium tumefaciens* as 5'-*Eco*R I/3'-*Bam*H I fragments. For this construct preparation, the patatin promoter and nos 3'UTR, separated by 5'-*Eco* RI/3'-*Bam* HI restriction sites, were cloned in pBluescript SK(+) and flanked by *Asc* I restriction sites. To introduce flanking 5'-*Eco* RI/3'-*Bam* HI, the crtB with transit peptide sequence was amplified by PCR using Phusion polymerase (New England Biolabs) with the oligonucleotides listed in Table S1. The two expression cassettes, crtB containing transit peptide sequence and DXS were introduced as *Asc* I fragments in tandem in the binary vector pKAN2, which is derived from the previously described pEC92 (Cahoon et al., 2006) by introduction of a *Mlu* I site upstream of the *Asc* I site. The *Mlu* I site was added to provide compatible cohesive ends for cloning of *Asc* I transgene cassettes. A two expression cassette

binary vector was prepared by cloning the patatin promoter-crtB-3' nos UTR cassette into the *Mlu* I site and the patatin promoter-DXS-3' nos UTR cassette into the *Asc* I site of pKAN2 to generate DXS//PS-pKAN2 (hereafter named DXS//PS). A second plant expression vector was prepared that contained only the crtB cassette cloned into the *Mlu*I site of pKAN2 to generate PS-pKAN2 (hereafter named as PS). The DXS//PS and the PS constructs were used to generate transgenic lines in cultivar 60444 background. For recovery of transgenic lines in TME 7 (Oko-iyawo), TME 7S, and TME 204 the same DXS and crtB expression cassettes described above were moved to a binary vector p5000 (Beyene et al., 2016a; Beyene et al., 2016b) to generate a construct named pEC20. The p5000 is a modified pCAMBIA2300. A large number of provitamin A accumulating lines in TME 204 cultivar were also generated using a construct designated p8001 (Chauhan et al., 2015) that combined expression cassettes of DXS and crtB for provitamin A accumulation and an inverted repeats of coat protein genes of *Ugandan cassava brown streak virus* (UCBSV) and *Cassava brown streak virus* (CBSV) fused in tandem. The p8108 was a variation of the pEC20 described above except that the nos terminator used in both crtB and DXS cassettes was replaced with a 425 bp homologues UTR obtained from the patatin type-I gene 3' UTR. The p8108 was used generate transgenic potato lines.

The plant expression vector containing the mutant cauliflower *Orange* (*Or*) genes was the same as previously described for potato carotene biofortification (Lu et al., 2006). The mutant *Or* gene in this vector is under control of the potato granule-bound starch synthase promoter. All binary vectors were electroporated into *Agrobacterium* strain LBA4404 and used for transformation of cassava cultivars.

Production of transgenic cassava plants and establishment in greenhouse and field

Transgenic lines were produced by *Agrobacterium*-mediated transformation of friable embryogenic callus produced from cassava cultivars 60444, TME 7S, TME 204 and Oko-iyawo (TME 7) (Chauhan et al., 2015; Taylor et al., 2012). Regenerated plants were tested for presence and expression of the target transgenes by PCR and RT-PCR respectively using primer listed in Table S1. Lines which tested positive for presence and expression of the transgenes were micropropagated established and grown under greenhouse as previously described (Beyene et

al., 2016a; Taylor et al., 2012). Plants were harvested and evaluated after 12-16 weeks. To establish plants in the field *in vitro*-derived plants were established in 50 mL Falcon tubes (Ogwok et al. 2012) and shipped to the University of Puerto Rico, Mayaguez, USA. Upon arrival plants were transferred to soil and acclimated for 4 weeks under greenhouse and another 4 weeks in screenhouse before establishment in the field.

Production of transgenic potato lines

In vitro stock plants were used as a source of material for transformation of *Solanum tuberosum* Desiree with *Agrobacterium tumefaciens* LBA4404 containing the p8108 construct according to methods described by Van Eck et al. (2007). The reported method has since been modified with the substitution of 300 mg/l timentin (Gold Biotechnology, Inc., St. Louis, MO) for carbenicillin. Transgenic lines were verified by PCR analysis for the presence of *nptII*. For PCR analysis DNA was isolated from leaf material by homogenization in a buffer (0.2M Tris, 0.25M NaCl, 25 mM EDTA, 5 mg/ml SDS) and precipitation in isopropanol. The resultant pellet was washed in 70% ethanol and air dried. Primers used for PCR detection of *nptII* are listed in Table S1. Ten *nptII*-positive lines were transferred to the greenhouse. For acclimation to greenhouse conditions, 6-week-old *in vitro* plants were removed from the selective rooting medium, the medium was washed from the roots, and the plants were placed in 4-inch plastic pots containing Metro-Mix 360 (Griffin Greenhouse Supply, Auburn, NY). The plants were immediately covered with small, transparent plastic containers as they were transferred to soil and kept in a growth chamber for 1 week. The containers were removed and the plants were kept in the chamber for one additional week before being transferred to a greenhouse where they were maintained at 20 to 22°C on a 16-hr photoperiod. Approximately 1 month after the acclimation period, plants were transferred to 3-gallon pots containing Metro-Mix 360. Tubers were harvested 14 weeks after plants were transferred to the greenhouse. Upon harvest tubers were separated into size categories (small, medium and large) and representative samples of tubers from medium and large size categorizes were peeled, chopped and immediately frozen for further analysis. Dry matter content, total carotenoid, starch, glucose, sucrose and fatty acids were determined following the same procedure described for cassava storage roots.

Field evaluation of transgenic cassava lines

Transgenic 60444 lines and TME 204 lines co-expressing *crtB* and *DXS* genes, were field tested at the Isabela Agriculture Research Station of the University of Puerto Rico, Mayaguez, Puerto Rico, USA under confined field trial conditions and were conducted under permit conforming to rules and regulation of the United State Department of Agriculture and the Puerto Rican Department of Agriculture. Four consecutive trials were conducted with transgenic 60444 lines planted in a randomized complete block design with 3-6 replicates and 8-10 plants/line/replicates. The initial trial was established with *in vitro* plants (IVP), while the subsequent three trials (SP1-3) were from planted using stem cuttings from the previous season consecutive stake cuttings of the two lines DXS//PS-20 and DSX//PS-37. All trials were surrounded by a row of non-transgenic plants and were harvested 12 months after planting (MAP). IVP trial consisted of 3 replicates with 8 plants per line while SP1, SP2 and SP3 trials consisted of 4, 5 and 6, replicates each, respectively. The TME 204 transgenic lines derived from the construct p8001 were established in the field in three batches. Each trial was established using IVP in a single row with 10 plants per line. The storage roots were harvested from 4 middle plants of each line at 12 MAP for analysis. Drip-irrigation was used in the dry season; otherwise the trials were rain-fed. All trials were fertilized approximately 30 days after planting with 10-10-10 (N-P-K) fertilizer. At harvest, the following agronomic properties were assessed: above ground weight (kg), below ground weight (kg), number of roots and root dry matter content (%). In each of the 4 trials involving 60444 transgenic lines 3 roots/line/replicate were chopped and lyophilized for DMC and total carotenoid analysis. Five storage roots, of approximately equal size, per line of SP2 trials were subjected to a more thorough analysis including total carotenoids, DMC, post-harvest deterioration (PPD). Samples from SP3 plants harvested at 12 MAP were used for transcriptome and metabolome analyses.

Fatty acid and triacylglycerol analyses

Approximately 100 mg of lyophilized cassava storage root flour was extracted in 3 mL of chloroform:methanol (1:2 v/v) supplemented with 500 µg of triheptadecanoin (17:0-triacylglycerol, Nu Chek Prep) as an internal standard in a 13 X 100 mm glass screw cap tube. Following 30 min of incubation with agitation on a nutating mixer, 1 mL of chloroform and 1.8

mL of water was added to each sample and mixed thoroughly. Following centrifugation (1,000 *g* for 5 min), the lower lipid phase was transferred to another glass tube and dried under nitrogen. The lipid extract was resuspended in 100 µL of chloroform:methanol (6:1 v/v), and 25 µL of the extract dried under nitrogen and transesterified in 1 ml of 2.5% sulfuric acid/methanol (v/v) and 250 µL of toluene (Msanne et al., 2012) for measurement of total fatty acids. The remainder of the extract (75 µL) was dried under nitrogen and redissolved in 1 mL of heptane for purification of triacylglycerols using a procedure similar to that previously described (Zhu et al., 2016). The extract was applied to a 3 ml Supelco LC-Si solid phase extraction column equilibrated in heptane. The column was eluted with 500 µl of heptane, followed by 1 mL of heptane:diethyl ether (95:5 v/v). The column was then eluted with 2.5 mL of heptane:diethyl ether (80:20 v/v) and collected in a glass screw cap test tube. This fraction, containing eluted triacylglycerols, was dried under nitrogen and transesterified in 1 mL of 2.5% sulfuric acid/methanol (v/v) and 250 µl of toluene. Samples for transesterification were heated at 95 °C for 30 min in glass test tubes capped under nitrogen. The resulting fatty acid methyl esters were recovered and analyzed by gas chromatography with flame ionization detection as described (Msanne et al., 2012). Fatty acid methyl esters were quantified in the total lipid and triacylglycerol fractions by comparison of sample peak areas relative to that of methyl heptadecanoic acid from the internal standard.

HPLC conditions for carotenoid analyses

Analyses were conducted using an Agilent 1200 HPLC with a binary pump and detection by absorbance at 455 nm using a diode array detector. Separation of carotenoid species was achieved using a C30 ProntoSIL column (250 mm length, 4.6 mm inner diameter, 5 µm particle size) or a C30 Dionex Acclaim column (150 mm length, 4.6 mm inner diameter, 3 µm particle size) with an isocratic solvent system consisting of 80% methanol/20% methyl-*tert*-butyl ether at a flow rate of 1.4 ml/min, essentially as described (Rodriguez-Amaya and Kimura, 2004).

References

Beyene, G., Chauhan, R.D., Ilyas, M., Wagaba, H., Fauquet, C.M., Miano, D., Alicai, T. and Taylor, N.J. (2016a) A Virus-Derived Stacked RNAi Construct Confers Robust Resistance to Cassava Brown Streak Disease. *Front. Plant Sci.* **7**, 2052.

Beyene, G., Chauhan, R.D., Wagaba, H., Moll, T., Alicai, T., Miano, D., Carrington, J. and Taylor, N.J. (2016b) Loss of CMD2-mediated resistance to cassava mosaic disease in plants regenerated through somatic embryogenesis. *Molecular plant pathology* **17**, 1095-1110.

Cahoon, E.B., Dietrich, C.R., Meyer, K., Damude, H.G., Dyer, J.M. and Kinney, A.J. (2006) Conjugated fatty acids accumulate to high levels in phospholipids of metabolically engineered soybean and Arabidopsis seeds. *Phytochemistry* **67**, 1166-1176.

Cahoon, E.B. and Shanklin, J. (2000) Substrate-dependent mutant complementation to select fatty acid desaturase variants for metabolic engineering of plant seed oils. *Proceedings of the National Academy of Sciences of the United States of America* **97**, 12350-12355.

Chauhan, R.D., Beyene, G., Kalyaeva, M., Fauquet, C.M. and Taylor, N. (2015) Improvements in Agrobacterium-mediated transformation of cassava (*Manihot esculenta* Crantz) for large-scale production of transgenic plants. *Plant Cell Tiss. Org.* **121**, 591-603.

Lu, S., Van Eck, J., Zhou, X., Lopez, A.B., O'Halloran, D.M., Cosman, K.M., Conlin, B.J., Paolillo, D.J., Garvin, D.F., Vrebalov, J., Kochian, L.V., Kupper, H., Earle, E.D., Cao, J. and Li, L. (2006) The cauliflower Or gene encodes a DnaJ cysteine-rich domain-containing protein that mediates high levels of beta-carotene accumulation. *Plant Cell* **18**, 3594-3605.

Msanne, J., Xu, D., Konda, A.R., Casas-Mollano, J.A., Awada, T., Cahoon, E.B. and Cerutti, H. (2012) Metabolic and gene expression changes triggered by nitrogen deprivation in the photoautotrophically grown microalgae Chlamydomonas reinhardtii and Coccomyxa sp. C-169. Phytochemistry 75, 50-59.

Ogwok, E., Odipio, J., Halsey, M., Gaitan-Solis, E., Bua, A., Taylor, N.J., Fauquet, C.M. and Alicai, T. (2012) Transgenic RNA interference (RNAi)-derived field resistance to cassava brown streak disease. *Molecular plant pathology* **13**, 1019-1031.

Rodriguez-Amaya, D.B. and Kimura, M. (2004) HarvestPlus handbook for carotenoid analysis. Washington DC:International Food Policy Research Institute.

Taylor, N., Gaitan-Solis, E., Moll, T., Trauterman, B., Jones, T., Pranjal, A., Trembley, C., Abernathy, V., Corbin, D. and Fauquet, C.M. (2012) A High-throughput platform for the production and analysis of transgenic cassava (Manihot esculenta) plants. *Trop. Plant Biol.* **5**, 127-139.

Van Eck, J., Conlin, B., Garvin, D.F., H, M., DA, N. and CR, B. (2007) Enhancing Beta-carotene content in potato by RNAi-mediated silencing of the Beta-carotene hydroxylase gene. *Amer. J. Potato Res.* **84**, 331.

Zhu, L.H., Krens, F., Smith, M.A., Li, X., Qi, W., van Loo, E.N., Iven, T., Feussner, I., Nazarenus, T.J., Huai, D., Taylor, D.C., Zhou, X.R., Green, A.G., Shockey, J., Klasson, K.T., Mullen, R.T., Huang,

B., Dyer, J.M. and Cahoon, E.B. (2016) Dedicated Industrial Oilseed Crops as Metabolic Engineering Platforms for Sustainable Industrial Feedstock Production. *Scientific Reports* **6**, 22181.

Table S1. List of genes and primer pairs used for cloning, detection and expression of the transgenes and expression analysis of cassava starch biosynthetic genes

Primer Name	Sequence	Target gene	Purpose	Reference
crtB-EcoRI	TTTGAATTCAAATGGCCATGAAACTGAATGC	crtB	Introduction of restriction site	This study
crtB-BamI	TTTGGATCCATCACAAGGGTCTCTGCCACAGATG			This study
Pri19	CGATGATAGACCAAGTTGCTTC	DXS	Transgene presence and expression	This study
Pri20	AAGACCGGCAACAGGATTCA			
Pri21	AGGGAGGCACAGTATTCACAAT	crtB	Transgene presence and expression	This study
Pri22	AAGACCGGCAACAGGATTCA			
Pri323	GCATAAGACCAGCCTCGGCA	DXS	RT-qPCR	This study
Pri324	TGGTTTGTTGGATGGCAAGC			
Pri325	CTTGCAGCCAACAAAAGCGT	crtB	RT-qPCR	This study
Pri326	TGGTGTGAAGGTAGAGCAAGCC			
Pri837	TGCAAGGCTCACACTTTCATC	PP2A	Reference gene for RT-qPCR	Moreno et al. (2011)
Pri838	CTGAGCGTAAAGCAGGGAAG	(Manes.09G039900.1)		
Pri960	CACTGATGAGCCTCCAAAGTTTGA	MeAGPL3	RT-qPCR	This study
Pri961	AATCCGGCACTTATCCATTTTGG	(Manes.11G085500.1)		
Pri964	TTGTCATGGAAGGGACCAGCCA	MeGBSS1	RT-qPCR	This study
Pri965	CGTGGGAACGTTCTCCTTAGCA	(Manes.02G001000.1)		
Pri968	GGAGTGTGTTGGATGGGATCACA	MeSS1	RT-qPCR	This study
Pri969	TCCAATCAATGGACACATAGGCC	(Manes.01G184000.1)		
nptII-FF	GGCTGGAGAGGCTATTC	nptII	Detection of transgene in potato	This study
nptII-R	GGAGGCGATAGAAGGCG			

Table S2. Selected differentially expressed genes associated with generation of carbon precursors for fatty acid biosynthesis (pyruvate dehydrogenase, acetyl-CoA carboxylase), *de novo* fatty acid biosynthesis (β-ketoacyl acyl carrier protein synthase I, III), or fatty acid storage as triacylglycerols (diacylglycerol acyltransferases 1, 2, 3) between provitamin A accumulating lines (DXS//PS-20 and DXS//PS-37) and wild-type controls of cassava storage roots at 12 months after planting. Fold-changes are presented relative to the wild-type control. Cassava orthologs of Arabidopsis genes are presented with TAIR IDs.

**Enzymes**	**Arabidopsis thaliana gene abbrev.**	**Annotation**	**TAIR-ID**	**Cassava**	**Fold-change (Line-37)**	**Fold-change (Line-20)**
**Pyruvate Dehydrogenase complex**	α-PDH	Pyruvate Dehydrogenase alpha subunit	At1g01090	Manes.05G016500.1	-0.312326681	-0.283099065
	β-PDH	Pyruvate Dehydrogenase beta subunit	At2g34590	Manes.17G071300.1	-0.368791002	-0.601230188
				Manes.15G122600.1	0.180260367	0.023568843
	EMB3003	Dihydrolipoamide Acetyltransferase	At1g34430	Manes.02G190800.1	-0.639978682	-0.598299713
	LTA2	Dihydrolipoamide Acetyltransferase	At3g25860	Manes.14G100900.1	-0.025945815	-0.066539896
**Acyl-CoA Carboxylase**	BCCP2 (cac1b)	Biotin Carboxyl Carrier Protein	AT5G15530.1	Manes.01G097500.1	-0.960441258	-0.721004718
				Manes.02G053800.1	NA	NA
	BCCP1 (cac1a)	Biotin Carboxyl Carrier Protein	AT5G16390.1	Manes.12G004000.1	-0.154964855	-0.200452146
				Manes.13G004100.1	-0.269904278	-0.136961022
	α-CT	carboxyltransferase alpha subunit of acetyl-CoA carboxylase	AT2G38040.1	Manes.08G087100.1	0.358776811	0.58283664
				Manes.09G101700.1	1.743544215	2.709765184
	β-CT	Carboxyltransferase beta Subunit of Heteromeric ACCase	AtCg00500	**Not found**		
**Ketoacyl-ACP Synthase I**	KASI	Beta-ketoacyl-[acyl carrier protein] synthase I	AT5G46290.1	Manes.02G007700.1	-0.717604702	-0.829934581
				Manes.15G120000.1	-0.282423353	-0.123211039
**Ketoacyl-ACP Synthase III**	KAS III	Beta-ketoacyl-[acyl-carrier-protein] synthase III	AT1G62640.2	Manes.02G036200.1	-0.25472249	-0.269891915
				Manes.01G076900.1	0.203178669	0.188985188
**Diacylglycerol acyltransferase**	DGAT1	Diacylglycerol acyltransferase 1	At2g19450.1	Manes.16G100000.1	0.951882235	0.579467504
			-	Manes.03G036000.1	0.819998978	0.316086018
	DGAT2	Diacylglycerol acyltransferase 2	At3g51520.1	Manes.01G158200.1	0.170181166	0.185068665
			-	Manes.01G158100.1	NA	NA
			-			
	DGAT3	Diacylglycerol acyltransferase 3	At1G48300.1	Manes.01G234700.1	0.046424558	0.060747278

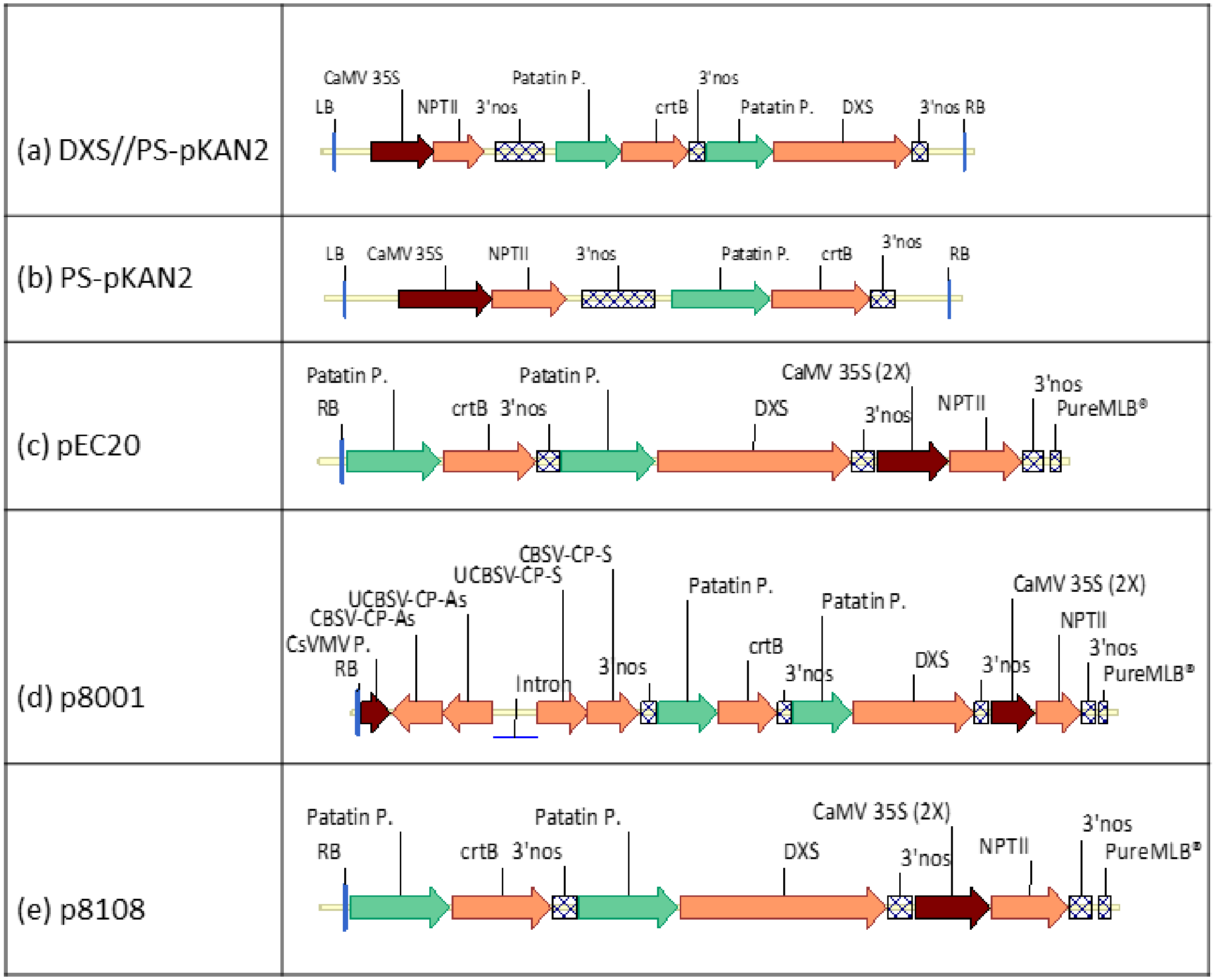

Figure S1. Maps of the T-DNA regions of the different constructs used for provitamin A enhancement of cassava storage roots. (a) T-DNA harboring expression cassettes of *crtB* and *DXS* in pKAN2 based binary vector named DXS//PS, (b) T-DNA harboring expression cassette of *crtB* in pKAN2 based binary vector named PS, (c) T-DNA harboring expression cassettes of *crtB* and *DXS* in p5000 based binary vector named pEC20 and (d) T-DNA harboring expression cassettes of *crtB*, *DXS* and an inverted repeat of coat protein genes from *Cassava brown streak virus* and *Ugandan cassava brown streak virus* fused in tandem in p5000 based binary vector named as p8001 and (e) T-DNA harboring expression cassettes of *crtB* and *DXS* each with patatin promoter and patatin 3'UTR in p5000 based binary vector named p8108. The p5000 binary vector modified from pCAMBIA2300.

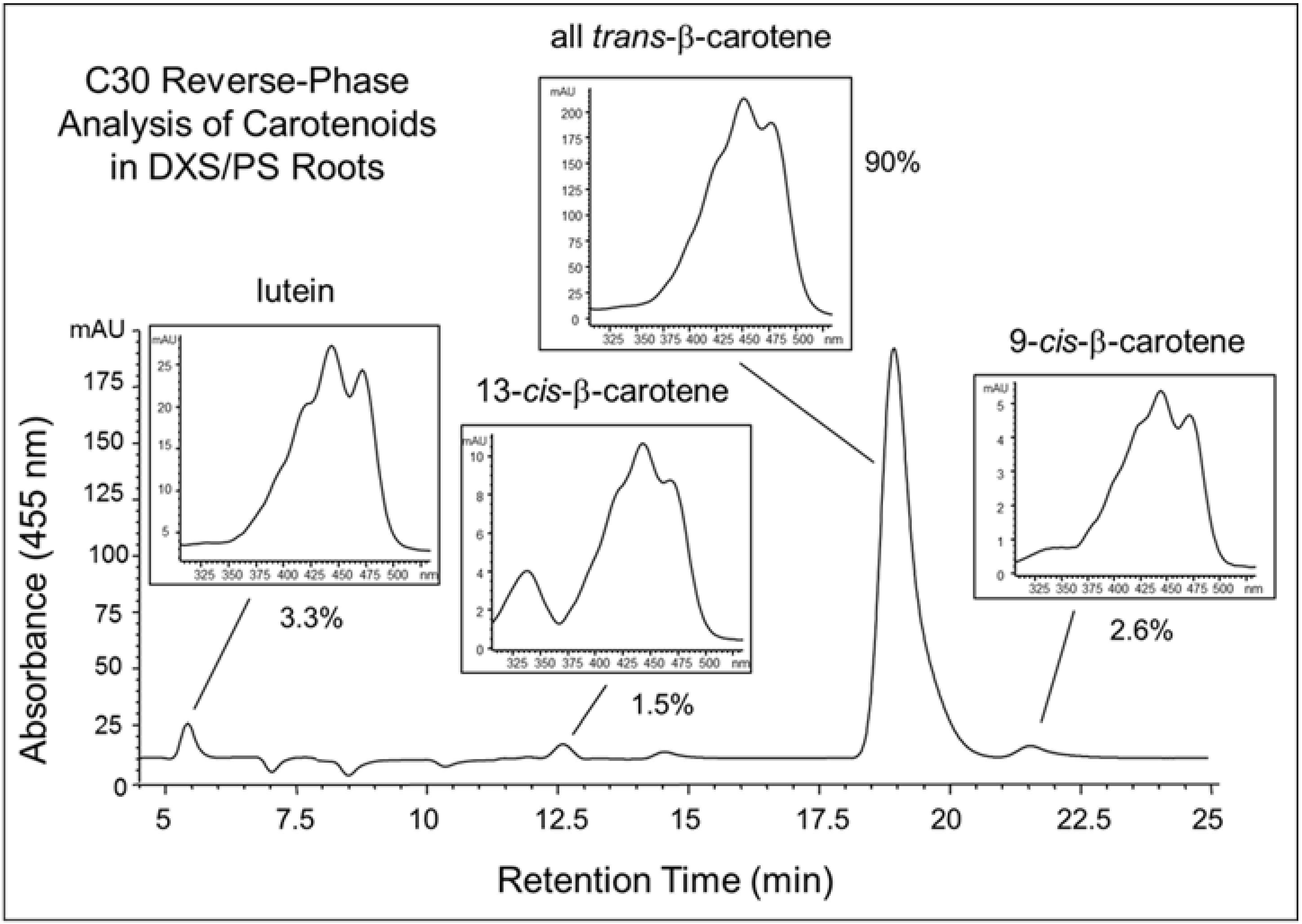

Figure S2. Representative chromatogram from the HPLC analysis of storage roots from DXS//PS engineered plants. Analyses were conducted essentially as described in HarvestPlus Handbook for Carotenoid Analysis (Rodriguez-Amaya and Kimura, 2004) using a C30 reverse phase column, an isocratic solvent system of 80:20 methanol:*t*-butyl methyl ether (v/v), and detection by absorbance at 455 nm using a diode array detector. Absorbance scans of major peaks from 300 nm to 700 nm were conducted for structural conformation of carotenoid species as described in the HarvestPlus Handbook for Carotenoid Analysis.

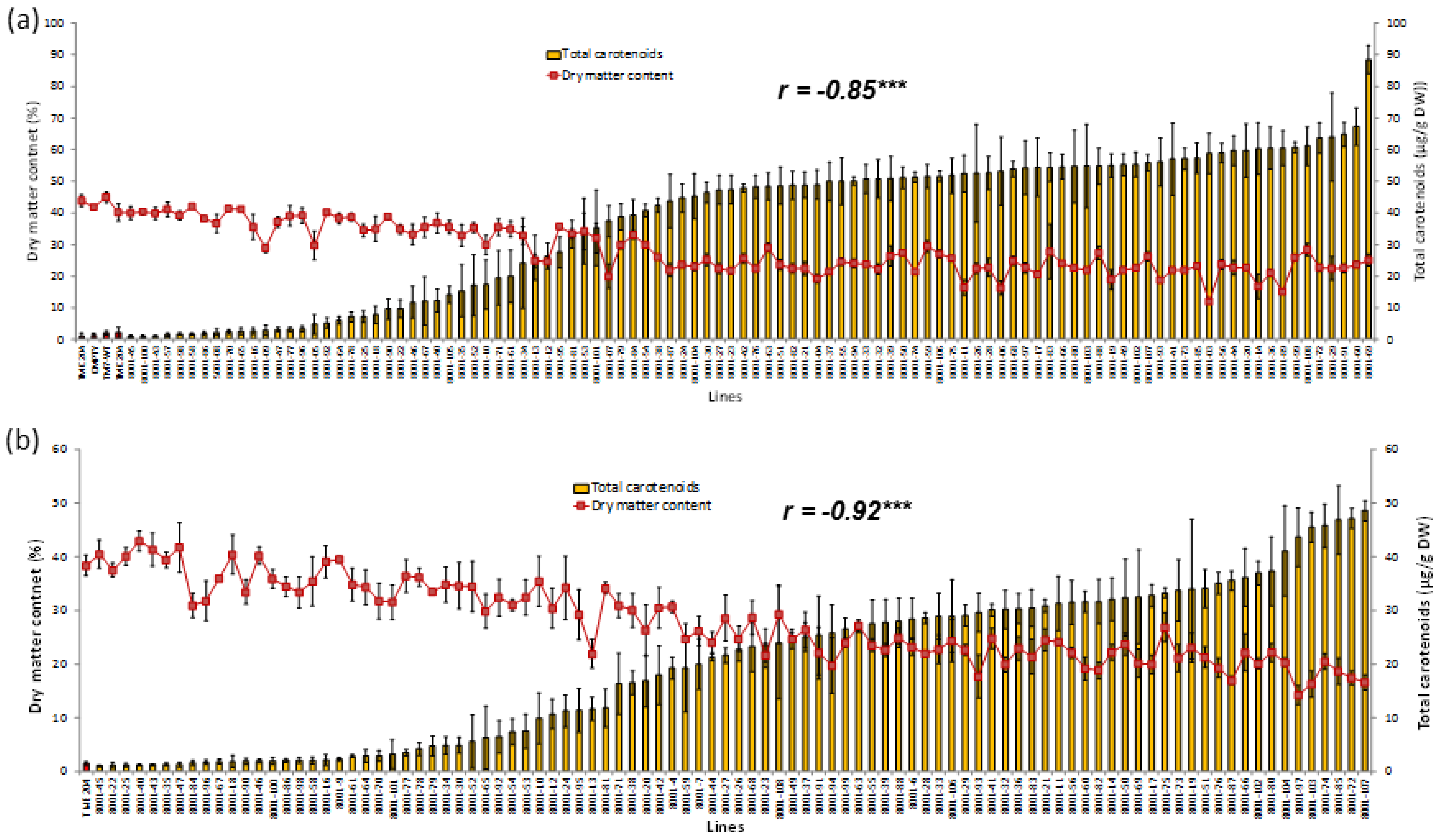

Figure S3. Dry matter and total carotenoid content of transgenic p8001 TME 204 cassava lines. (a) Greenhouse grown for 16 weeks (b) Grown under confined field trial in Puerto Rico for 12 months. The transgenic lines were generated using a construct p8001 that has expression cassettes of *crtB* and *DXS* genes for carotenoids and an inverted repeat of coat protein genes from *Cassava brown streak virus* and *Ugandan cassava brown streak virus* fused in tandem. Bars are SD.

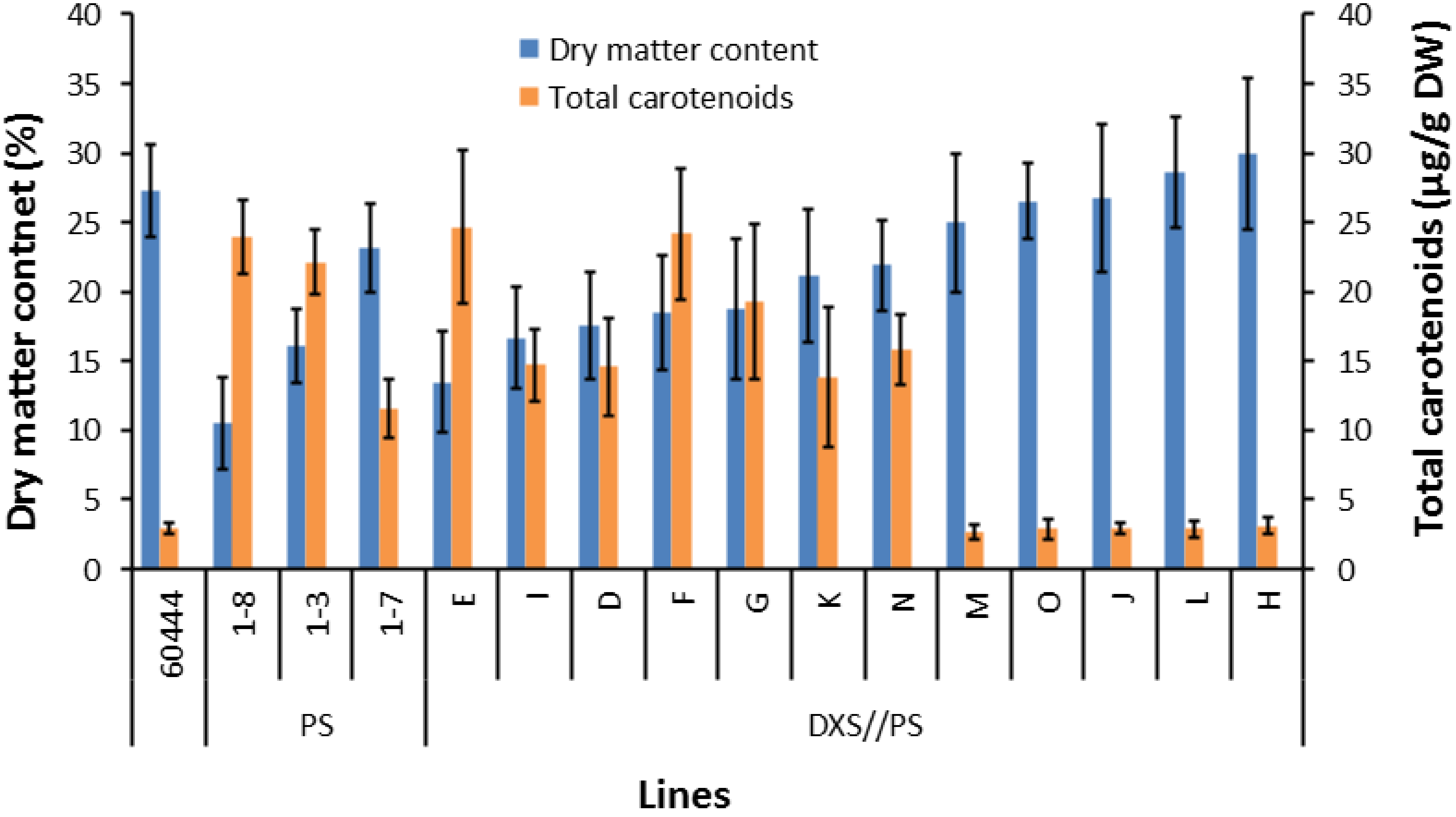

Figure S4. Dry matter and total carotenoid content of transgenic lines expressing *crtB* alone (PS-lines) or co-expressing crtB and DXS (DXS//PS-lines). Transgenic PS and DXS//PS-lines and non-transgenic control cultivar 60444 were grown in replicated plots (4 replicates of 6 plant each) under confined field trial condition in Puerto Rico. Four plants per plot were harvested from the for evaluation of dry matter content and total carotenoids at 12 months after planting. Bars are SD.

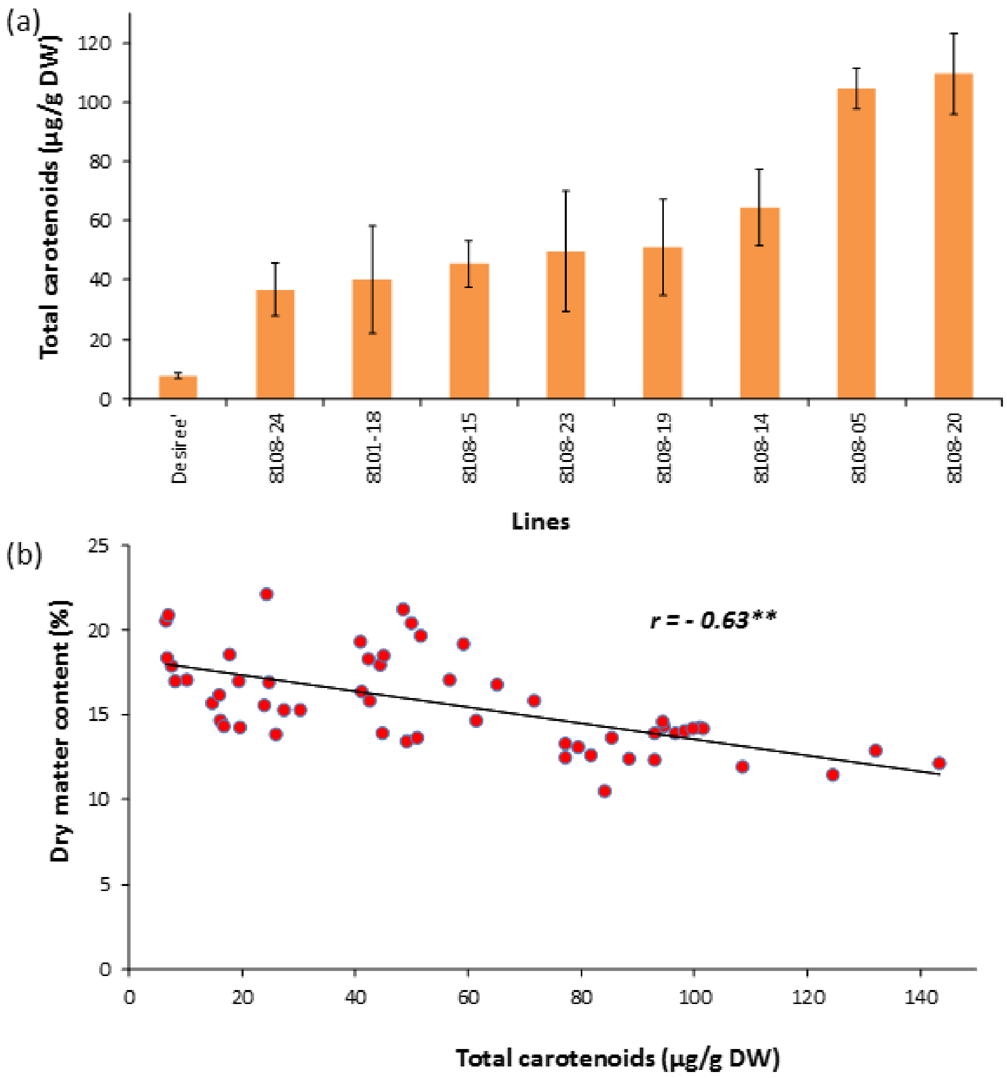

Figure S5. Total carotenoid and dry matter content in transgenic p8108 potato lines co-expressing *crtB* and *DXS*. (a) total carotenoids and, (b) correlation between total carotenoids and dry matter content. Transgenic potato lines were generated using p8108 that harbors the *crtB* and *DXS* transgenes each driven by patatin type I promoter and the homologues untranslated region for transcript termination. Data were generated from tubers harvested at 14 weeks after planting in the greenhouse. Bars are SE of 4-6 biological replicates per line.
